# Dynamic Regulation of Synaptopodin and the Axon Initial Segment in Retinal Ganglion Cells During Postnatal Development

**DOI:** 10.3389/fncel.2019.00318

**Published:** 2019-07-30

**Authors:** Annabelle Schlüter, Sabrina Rossberger, Dominik Dannehl, Jan Maximilian Janssen, Silke Vorwald, Janina Hanne, Christian Schultz, Daniela Mauceri, Maren Engelhardt

**Affiliations:** ^1^Institute of Neuroanatomy, Center for Biomedical Research and Medical Technology, Medical Faculty Mannheim, Heidelberg University, Mannheim, Germany; ^2^Department of Neurobiology, Interdisciplinary Center for Neurosciences, Heidelberg University, Heidelberg, Germany; ^3^Kirchhoff-Institute for Physics, Applied Optics, Heidelberg University, Heidelberg Germany; ^4^Abberior Instruments GmbH, Heidelberg, Germany

**Keywords:** axon initial segment, cisternal organelle, synaptopodin, retinal ganglion cell, visual deprivation

## Abstract

A key component allowing a neuron to function properly within its dynamic environment is the axon initial segment (AIS), the site of action potential generation. In visual cortex, AIS of pyramidal neurons undergo periods of activity-dependent structural plasticity during development. However, it remains unknown how AIS morphology is organized during development for downstream cells in the visual pathway (retinal ganglion cells; RGCs) and whether AIS retain the ability to dynamically adjust to changes in network state. Here, we investigated the maturation of AIS in RGCs during mouse retinal development, and tested putative activity-dependent mechanisms by applying visual deprivation with a focus on the AIS-specific cisternal organelle (CO), a presumed Ca^2+^-store. Whole-mount retinae from wildtype and Thy1-GFP transgenic mice were processed for multi-channel immunofluorescence using antibodies against AIS scaffolding proteins ankyrin-G, βIV-spectrin and the CO marker synaptopodin (synpo). Confocal microscopy in combination with morphometrical analysis of AIS length and position as well as synpo cluster size was performed. Data indicated that a subset of RGC AIS contains synpo clusters and that these show significant dynamic regulation in size during development as well as after visual deprivation. Using super resolution microscopy, we addressed the subcellular localization of synpo in RGC axons. Similar to cortical neurons, RGCs show a periodic distribution of AIS scaffolding proteins. A previously reported scaffold-deficient nanodomain correlating with synpo localization is not evident in all RGC AIS. In summary, our work demonstrates a dynamic regulation of both the AIS and synpo in RGCs during retinal development and after visual deprivation, providing first evidence that the AIS and CO in RGCs can undergo structural plasticity in response to changes in network activity.

## Introduction

In the retina, visual processing relies on a chain of neurons that transmit information from the outmost photoreceptor layer to the innermost retinal ganglion cell (RGC) layer (Masland, 2012). RGCs convey information via higher order nuclei in the visual pathway to the primary visual cortex (V1) and associated regions. During development, V1 undergoes defined critical periods of cortical plasticity, which shape and refine the mature visual map (reviewed in Hensch, 2004). These critical periods are defined by activity patterns across the visual pathway, and lack of neuronal activity and visual input leads to significant alterations of the final network configuration in V1 (reviewed in Hensch, 2005). While the role of somatodendritic plasticity in this context has been studied extensively (Levelt and Hubener, 2012), it has only recently been acknowledged that axonal plasticity may play an equally important role for the development of sensory cortices (reviewed in Jamann et al., 2017).

The axon initial segment (AIS) is a structurally and molecularly unique axonal domain (Huang and Rasband, 2018; Leterrier, 2018) and is the site of action potential initiation (Kole and Stuart, 2012). Recent work has suggested that it is linked to regulating neuronal excitability on a single cell level, most likely via homeostatic mechanisms (reviewed in Wefelmeyer et al., 2016; Engelhardt et al., 2019). This concept is based on a growing body of evidence from *in vitro* and *in vivo* studies and across multiple species showing that the AIS contributes to cellular excitability by increasing or decreasing its length or distance to the soma in response to changes in network activity (reviewed in Yamada and Kuba, 2016; Jamann et al., 2018). These events are thought to be a direct result of changes in synaptic drive either during development or in the mature network: Longer AIS reflect a decrease in synaptic drive, for example after sensory deprivation, and facilitate action potential generation, whereas shorter or distally relocated AIS result from an increase in synaptic drive and are correlated with decreased excitability (reviewed in Jamann et al., 2018).

In work leading to the present study, we described the activity-dependent structural maturation of the AIS in V1 pyramidal neurons, which undergo distinct periods of length maturation during the postnatal phase until closure of the critical period of cortical plasticity in mice around P35 (Gutzmann et al., 2014). Visual deprivation led to a significant modulation of this maturation period; in fact, V1 pyramidal neurons were no longer able to reach mature AIS lengths (Gutzmann et al., 2014). The underlying mechanisms that regulate AIS plasticity, in particular length and location changes, remain largely unknown. Recent evidence indicates that intraaxonal Ca^2+^ levels may play an important role for AIS plasticity and considering the importance of Ca^2+^ currents for the generation and timing of action potentials (Bender and Trussell, 2009), this seems intuitive. Our previous work highlighted a role for the cisternal organelle (CO) in AIS plasticity in V1 pyramidal neurons (Schlüter et al., 2017). The CO consists of stacks of smooth endoplasmic reticulum and is a putative regulator of Ca^2+^-storage and release in the AIS (Benedeczky et al., 1994; Bas Orth et al., 2007). The structural integrity of the CO is organized by the actin-binding protein synaptopodin (synpo; Bas Orth et al., 2007), which therefore is an excellent molecular marker of this intraaxonal compartment (Sanchez-Ponce et al., 2011; Schlüter et al., 2017).

Currently, it is unclear whether RGC AIS constitute a group of neurons similar to cortical pyramidal cells in terms of AIS morphology, maturation and plasticity, and whether putative AIS plasticity in RGCs could be partially regulated by the dynamic remodeling of the CO. In the present study, we therefore investigated the structural maturation of AIS length and synpo expression in RGCs during retinal development and in dark-reared mice, utilizing a combination of multi-channel immunofluorescence, confocal and super resolution microscopy as well as morphometrical analysis. Our data indicate that RGC AIS share a common nanostructure with other excitatory neurons of the visual pathway. Furthermore, RGC AIS develop with similar activity-dependent profiles as shown for V1: Both the AIS and synpo in RGCs undergo periods of dynamic remodeling during retinal development and after visual deprivation, indicating that organelles in the AIS of RGCs can undergo structural plasticity in response to changes in network activity and might therefore impact cellular function during visual processing.

## Materials and Methods

### Animals

The following mouse and rat strains were used: wildtype C57BL/6JRj mice (Janvier Labs, France), B6.Cg-Tg(Thy1-EGFP)MJrs/J mice (own colony at Heidelberg University), and wildtype RjHan:SD rats (Janvier Labs, France). Animals of mixed gender from C57BL/6JRj strain, Tg(Thy1-EGFP)MJrs/J strain, and RjHan:SD strain were maintained with food and water *ad libitum* on a regular 12 h light/dark cycle. C57BL/6JRj wildtype mice were used for deprivation studies, reared on a 24 h dark cycle as described below, and for the postnatal development study (ages P10 to *P* > 55). For morphometrical studies of AIS and synpo cluster localization, adult (*P* > 55) wildtype mice and rats as well as Thy1-EGFP mice were used.

### Developmental Study

For the analysis of AIS length and synpo expression in RGC AIS during retinal development, a total of 6 wildtype mice were analyzed in each of the following age groups: P10, P15, P21, P28, P35, P > 55 (two retinae per mouse, at least 100 AIS per animal ≜ at least 600 AIS/group). For the analysis of AIS in Thy1-EGFP mice, a total of 5 adult (*P* > 55) mice and a total of 5 control (*P* > 28) mice were used. For super resolution imaging of the AIS scaffold and synpo clusters in AIS of RGCs, a total of 5 adult (*P* > 55) wildtype rats and mice were analyzed. Thirty AIS were examined in total. A summary of all experimental groups is given in Table 1.

**TABLE 1:** Experimental and control groups used in the current study with indication of age of animal, mouse strain, period of visual deprivation, and treatment of tissue for immunofluorescence.

**Strain**	**Control**	**Visual deprivation**	**Fixation (4% PFA)**
**Wildtype mice**			
C57Bl/6JRj (*n* = 6)	P10, P15, P21		Perfusion
	P28	From birth (P0–28)	Perfusion
	P35	From birth (P0–35)	Perfusion
	*P* > 55		Perfusion
**Thy1-EGFP mice**	*P* > 28	From birth (P0–28)	Perfusion
B6.Cg-Tg(Thy1 EGFP)MJrs/J (*n* = 5)	*P* > 55		Perfusion
**Wildtype rats**			
RjHan:SD (*n* = 5)	*P* > 55		Perfusion

### Visual Deprivation

Wildtype and Thy1-EGFP mice were kept in completely dark cages with food and water *ad libitum*. Total absence of light was controlled by exposure of photographic paper located in the cages. Groups of 6 (wildtype) and 11 (Thy1-EGFP) mice each were reared in complete darkness from P0–28 and P0–35, respectively. Analyses then proceeded immediately after the period of visual deprivation. Data from the developmental study (P28 and 35, wildtype; *P* > 28, Thy1-EGFP) served as controls for deprivation experiments. All experimental groups are summarized in Table 1.

### Tissue Preparation

Mice were exsanguinated with 0.9% saline under deep anesthesia with ketamin (120 mg/kg BW)/xylazine (16 mg/kg BW). Animals were then perfused transcardially with ice-cold 4% paraformaldehyde (PFA) in 1x PBS. Whole retinas were extracted and processed as follows: Eyes were enucleated with fine curved forceps and were transferred into a 5 cm culture dish containing ice-cold 1x PBS. Retinae were dissected under a stereomicroscope with an internal light source. A hole was cut into the eye at the corneal limbus by using a pair of fine spring scissors. After a circumferential incision along the limbus, the cornea, iris, vitreous body, and lens were removed *in toto*. The retina was extracted from the eye cup. Retinae were fixed in 4% PFA for 10 min and were washed three times in 1x PBS before further processing.

### Immunofluorescence

Double and triple immunofluorescence was performed on free-floating whole retinae as described previously (Schlüter et al., 2019). Retinae were blocked for 4-5 h (in 0.5% Triton X-100, 0.2% bovine serum albumin and 0.02% sodium azide in 1x PBS) at 4°C. Primary antibodies were diluted in dilution buffer (1% Triton X-100, 10% fetal calf serum and 0.02% sodium azide in 1x PBS) according to previously validated concentrations (Gutzmann et al., 2014; Schlüter et al., 2017). Retinae were incubated with primary antibodies for 24 h at 4°C. Afterward, tissue was washed three times in 1x PBS for 15 min each. Retinae were then incubated with fluorophore-conjugated secondary antibodies diluted in dilution buffer for 24 h at 4°C, followed by additional washing steps (3x in 1x PBS, 15 min each). Prior to mounting, retinae were fixed for 10 min in 4% PFA and washed in 1x PBS (2x for 5 min). Tissue was then cut from the rim to 1/3 of the radial length and was flat-mounted on slides. All performed stainings were accompanied by negative controls, in which omitting the primary antibody completely abolished all stainings. For confocal and STED microscopy, retinae were embedded in Roti®-Mount FluorCare mounting medium or Mowiol (Carl Roth, Karlsruhe, Germany). For SIM and SMLM, retinae were embedded in ProLong^®^ Gold mounting medium (Thermo Fisher Scientific, Waltham, MA, United States) or switching buffer [10% dd H_2_O, 10% 1x PBS, 80% glycerol, 0.1M cysteamine, 100u glucose oxidase (Type VII from *Aspergillus niger*), 800u catalase (from Bovine liver), 0.1M D-(+)-Glucose]. For shift corrections (SIM and SMLM), 1 μl of FluoSpheres^®^ (FluoSpheres^®^ Fluorescent Color Kit, Carboxylate-Modified Microspheres, 0.04 μm, four colors; Thermo Fisher Scientific, Rockford, IL, United States) was transferred near the edge of the slide. Coverslips were sealed onto slides using transparent nail polish. All antibodies used in this study are summarized in Supplementary Table 1.

### Confocal Microscopy

Conventional laser scanning confocal microscopy was carried out using a C2 confocal microscope (Nikon, Alzenau, Germany; laser lines: 642, 543, and 488 nm), with a 60x objective (oil immersion, NA 1.4) and a SP5 confocal microscope Leica, Mannheim, Germany; Laser lines: 633, 561, 514, and 488 nm) with a 63x objective (oil immersion, NA 1.4), respectively. To increase the number of in-focus immunoreactive structures, stacks of images were merged into a maximum intensity projection and saved as jpeg and tif format. Thickness of single optical sections was 0.2 μm in stacks of 3–5 μm total depth. Confocal x-y-resolution was constantly kept at 0.21 μm per pixel. Images for qualitative analysis were evaluated and enhanced for contrast in Fiji (Image J) and Photoshop C5 (Adobe Systems, United States).

### Super Resolution Microscopy

Three different super resolution methods were applied to obtain a comprehensive understanding of the RGC AIS nanostructure.

Stimulated Emission Depletion (STED) imaging was performed using a STEDYCON (Abberior Instruments GmbH, Göttingen) with excitation lasers at 488, 561, and 640 nm, and a STED laser at 775 nm (maximum intensity 1.25 W; all lasers are pulsed with 40 MHz repetition rate). The STEDYCON was mounted on the camera port of an AxioImager.Z2 upright microscope (Zeiss, Jena, Germany), equipped with a 100x objective (alpha Plan-Apochromat, Oil, DIC, Vis, NA 1.46; Zeiss). The pinhole was set to 1.1 Airy units for 650 nm emission. Fluorescence was detected on avalanche photo diodes, with emission bands between 650–700 nm, 578–627 nm, and 505–545 nm, respectively. Data was stored in .obf format and exported as tif files for further analysis.

Correlative Structured Illumination Microscopy (SIM) and Single Molecule Localization Microscopy (SMLM) were performed using a custom-built microscope, which is described in detail in Rossberger et al. (2013). For acquisition, a high numerical objective (Leica HCX PL APO 100x/1.4 oil CS), a charge-coupled device-camera (Sensicam QE, PCO, Kelheim, Germany) and the following laser lines were used: 568 nm laser line (Coherent Sapphire 568 HP, 200 mW, Coherent, Dieburg, Germany) and 671 nm (VA-I-300-671, 300 mW, Beijing Viasho Technoloy Co. Ltd., Beijing, China). For each laser line, excitation and emission light were separated using an appropriate dichroic mirror (Di02-R568 and Q680LP, both Semrock, Rochester, NY, United States) and emission filter combination (BLP01-561, Semrock and LP XF 3104, Omega Optical, Olching, Germany).

Prior to SIM and SMLM acquisition, sections were scanned for correlated AIS-(568 nm) and Synpo-(647 nm) signal using the widefield mode of the microscope. For this purpose, the 568 nm excitation laser line was used in combination with an edge basic longpass emission-filter (BLP01-568R-25, Semrock, Rochester, NY, United States). The resulting widefield image (pixel size of 64.5 nm) shows an overlay of both color channels within one image and was used for shift corrections of the two color channels recorded separately in super resolution mode. Details of SIM and SMLM image acquisition are outlined in the Supplementary Methods.

### Morphometrical Analysis

We classified RGCs in Thy1-GFP mice where the entire cell could be visualized and measured soma size, dendritic tree diameter and stratification of dendrites into the inner plexiform layer (IPL) according to previously published guidelines (Sun et al., 2002b). We used parameters of soma and dendritic field size to classify RGCs into the different classes (A1–A2, B1–B4, C1–C6, D1–D2; O’Brien et al., 2014) as well as dendritic stratification into the IPL to further isolate A RGCs (RGC_A_) into ON- and OFF-ganglion cells (Anderson et al., 2016; Saha et al., 2016). Confocal z-stack images of entire RGCs were projected using AutoQuant X3 software (Media Cybernetics). The obtained three-dimensional images were turned 90 degrees to visualize the stratification of the dendritic tree of RGCs within the IPL (Supplementary Figure S1A). ON-ganglion cells were identified based on the ramification of their dendritic processes in sublamina b of the IPL whereas OFF-ganglion cells ramify in sublamina a of the IPL. In order to confine our data analysis to the RGC layer specifically and avoid any potential cross-contamination with cells or AIS from other retinal layers, confocal stacks were acquired exclusively within the ganglion cell layer (GCL) of the retina. The GCL could be identified clearly by NeuN immunostaining, nuclei labeling (TOPRO), and its location within 5–10 μm underneath the nerve fiber layer of the mouse retina. Thus, AIS of other retinal layers, such as AIS-like processes from AII amacrine cells located in the inner nuclear layer (Wu et al., 2011) were not included in our analysis. AIS in the GCL were selected only after clear identification of proximal and distal endpoints of immunoreaction to ankyrin-G (ankG) or βIV-spectrin could be traced across optical sections of a stack without any discontinuation or broad stratification into other layers, based on the 3D-projections created for the RGC classification (Supplementary Figure S1B). Overlapping AIS were not included in the analysis to avoid any potential interference of immunofluorescent signal with length and distance analysis.

AIS length and distance from the soma were obtained using a self-written program in Python (AISuite et al., unpublished; Hoefflin et al., 2017; Ernst et al., 2018). Briefly, in this application, the AIS is traced manually, beginning with the axon hillock and ending with the axon past visible AIS staining to exclude personal bias. The cut-off threshold for AIS length identification was set to 30% of the individual maximum intensity. Triplets of AIS signal values were analyzed to reduce the influence of upward outliers. The proximal and distal borders of the AIS were determined as the first, respectively the last triplet of signal values which were higher than the defined cut-off. The pixel difference between the proximal and distal end of the AIS was calculated and converted into length in μm taking the microscope’s calibration into account. AIS distance to the soma was determined by calculating the distance between the onset of the individual region of interest at the axon hillock and the estimated beginning of the AIS, using the same cut-off. All data were exported as HDF5 and Excel files for statistical analysis.

Periodicity of the AIS scaffold was analyzed similar to previously published methods (D’Este et al., 2015). Briefly, brightness and contrast were adjusted linearly for each STED and SMLM image. Fluorescence intensity plot profiles were measured using ImageJ along a 5 pixel wide line drawn along the AIS. For each AIS, a minimum of 3 regions were measured. The distances between interpeaks were determined using the peak analyze function in OriginPro software (Additive Friedrichsdorf, Germany).

### Synpo Expression Analysis

Number and size of synpo clusters were measured using a self-written macro in ImageJ as previously published (Schlüter et al., 2017). AIS containing synpo-positive immunofluorescence signals were manually outlined. Background signals beyond the AIS were eliminated. For cluster analysis, the “Color Threshold” option was used. Threshold level was set to 55 and minimum size of pixels was 5. Both values were kept constant during measurements. Synpo-positive clusters per AIS were defined by these parameters and were measured automatically for mean number and size (in pixels). For cluster sizes, mean pixel values were translated into μm^2^ (area [μm^2^] = area [pixels] × (microscope resolution)^2^).

### Image Analysis

For analysis of SIM data, nine images of conventional resolution for each z layer and each color channel were recorded. These images were reconstructed and deconvolved using custom developed software written in Matlab (Best et al., 2011). Image reconstruction resulted in one image for each layer within the z-stack and each color channel. For reconstruction of synpo clusters within RGC AIS, 3D SIM images were processed first by blind iterative deconvolution (theoretical PSF based on the optical properties of the microscope and the sample, 10x iteration) according to standard procedures in AutoQuant X3 (Media Cybernetics). Subsequently, to visualize x-y-z information about synpo cluster localization within the AIS, deconvolved files were reconstructed (surface) using Imaris 9.0 (Bitplane, Zurich) according to previously published protocols (Schlüter et al., 2017).

For analysis of SMLM data, single molecule positions of optically isolated molecules were determined by using custom software written in Python. Briefly, a Gaussian function was fitted to each single molecule signal and its center of mass was calculated. Center coordinates evaluated from all images of the sequence were transferred into one image, marking the position of one fluorophore. The mean position or localization accuracy was Δx = 10.6 nm, resulting in a position image. Images were rendered using a triangulation algorithm as previously published (Baddeley et al., 2010). The area spanned by three next neighbor fluorophore positions (triangle) was translated inversely into intensity values depending on the size of the area. High intensity values were translated into small distances between fluorophore positions and vice versa. Pixel size for visualization was either 5, 32.25, or 64.5 nm. Single molecule positions were randomly jittered to generate 100 slightly different images, which were overlaid afterward to smooth structure edges. Thus, single molecule positions within the images were transferred into an image showing structures in a more conventional sense.

### Statistical Analysis

Mean values of AIS length and position as well as size and number of synpo clusters per AIS were calculated in Excel (Microsoft). Standard deviation (SD) were calculated and mean values were plotted and analyzed in GraphPad Prism 7 software (GraphPad Software, San Diego, CA, United States). Values were tested for normal distribution by performing Shapiro–Wilk normality test (for testing mean values, *n* = 6) or D’Agostino and Pearson normality test (for testing single values, *n* > 600). Unpaired *t*-test or Mann–Whitney *t*-test was carried out for statistical comparison of two groups. One-way ANOVA, Kruskal–Wallis or two-way ANOVA test was applied for comparing three or more groups. *Post hoc* correction was performed by Tukeys or Sidak post test. Boxes extend from the 25th to 75th percentile. Error bars are drawn down to the minimum and up to the maximum value. Asterisks indicate significant differences (^*^p ≤ 0.05). For frequency distribution, bin centers were classified by dividing the entire range of values into a series of intervals. Values were then counted and assigned to each interval. For frequency distribution of ankG/β-spectrin periodicity, the entire range was 0–0.4 μm. Bin center steps were 0.02 μm. For frequency distribution of AIS length, the entire range was 10–55 μm. Bin center steps were 2 μm.

## Results

### AIS of Mature RGCs Are Characterized by a Distal Localization and a Defined Nanoscale Scaffold

**FIGURE 1:**
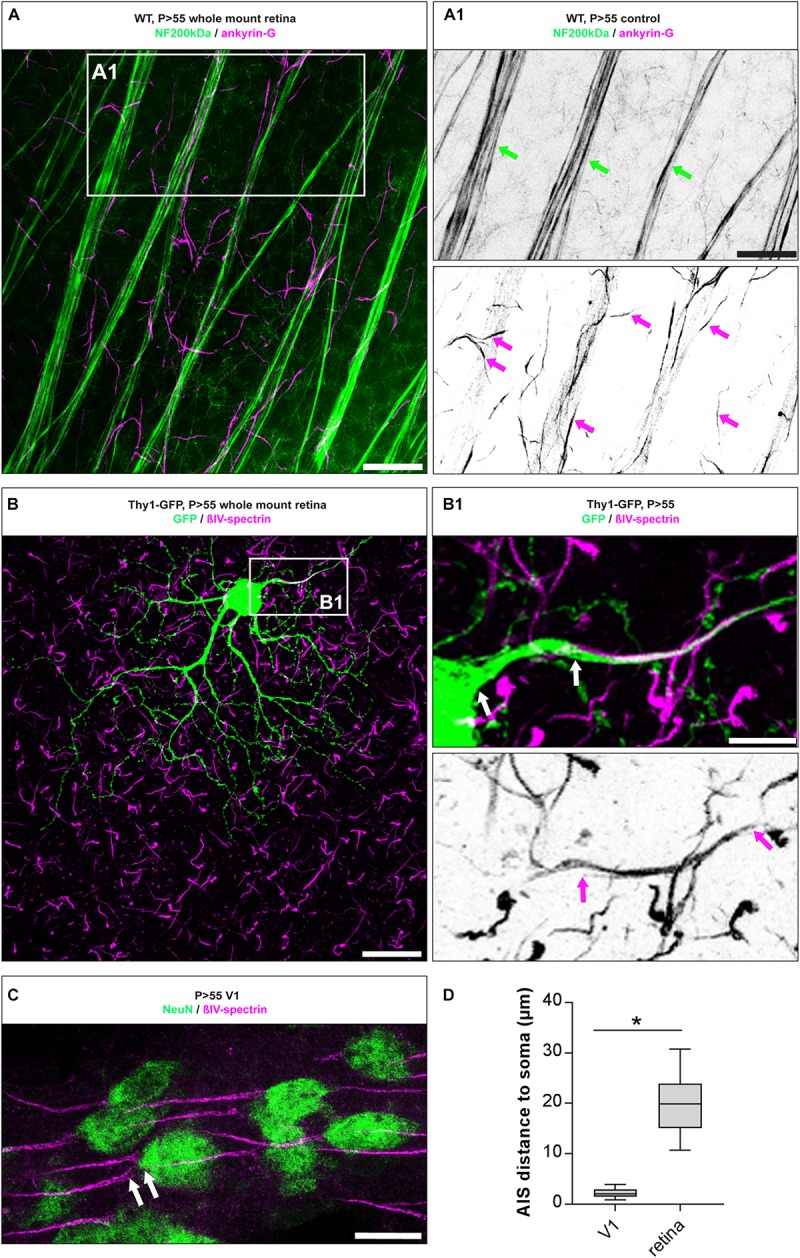
AIS morphology in mature RGC AIS. **(A)** Whole mount retina stained against neurofilament 200 kDa (NF200, green) and the AIS scaffolding protein ankyrin-G (ankG, magenta). **(A1)** Insert: magnification of double label against NF200 and ankG showing partial overlap in thick neuron tracks (green arrows), but exclusive label of ankG in the short, thin AIS of numerous RGCs (magenta arrows). **(B)** GFP-positive neuron in whole mount retina from a Thy1-EGFP animal. **(B1)** Insert: magnification of boxed region in **(B)**, indicating the gap between proximal AIS onset and the soma (upper panel, white arrows) and the length of the entire AIS (lower panel, magenta arrows). **(C)** Image of V1 cortical pyramidal neurons from adult mouse stained for NeuN (green) and βIV-spectrin (magenta), with only a small distance between soma and proximal AIS indicated by white arrows. **(D)** Quantification of the soma to AIS distance from V1 cortical neurons and RGCs. Unpaired *t*-test. ^*^p ≤ 0.05, *n* = 5 animals (at least 100 AIS per animal). Scale bar in **(A)** = 20 μm, **(A1)** = 10 μm, **(B)** = 30 μm, **(B1)** = 10 μm, **(C)** = 20 μm.

**FIGURE 2:**
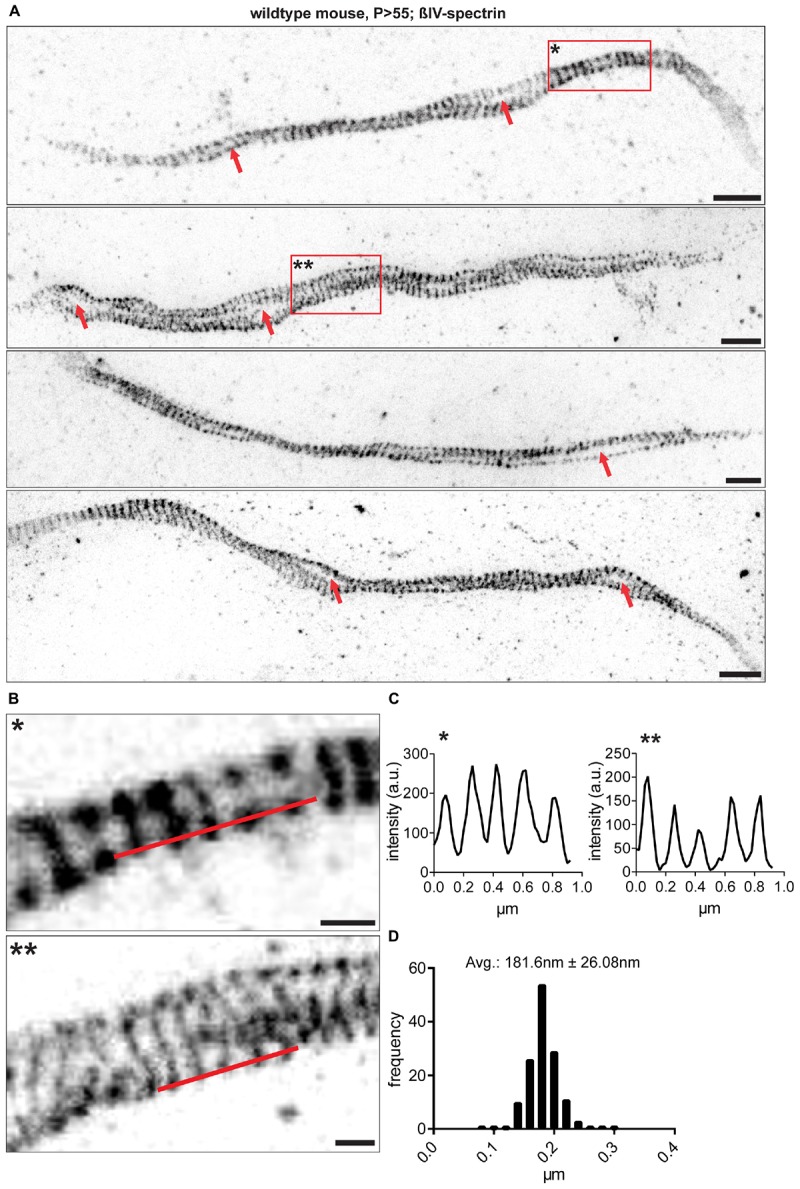
RGC AIS nanostructure imaged by STED microscopy. **(A)** Different samples from whole mount retina AIS immunofluorescence against βIV-spectrin. Data is shown after enhancement for contrast and colormap inversion. AIS show lattice-like structures and both presumptive ‘gaps’ and loops along their longitudinal axis (red arrows). **(B)** Inserts show zoom-ins of regions marked by red boxes with one and two asterisks in **(A)**. **(C)** Line profiles along red lines drawn in **(B)**. A periodically spaced intensity pattern is observed along the AIS. **(D)** Size frequency histogram of a single interpeak indicating the period scaffold to be spaced at approximately 180 nm. *n* = 5 animals (at least 100 AIS per animal). Scale bar **(A)** = 1 μm, **(B)** = 250 nm.

The morphology of RGC AIS in adult mouse retinae was characterized by immunofluorescence against typical AIS scaffolding proteins such as ankyrin-G (ankG) and βIV-spectrin, followed by confocal and super resolution microscopic qualitative analysis (Figures 1, 2). We found that both scaffolding proteins are expressed abundantly across all unmyelinated fiber tracts traversing the retina toward the optic disk and colocalize with neurofilament 200 kDa (Figure 1A). Individual RGC AIS are clearly distinguishable from the thicker fiber tracts based on their thin structure, increased ankG/βIV-spectrin expression, and decreased neurofilament 200kDa-immunoreaction (Figure 1A, magenta arrows). Using the transgenic Thy1 M-GFP mouse line, which sparsely labels RGCs under control of the Thy1 promoter in the retina (Feng et al., 2000), we observed a significant distance between the soma and proximal beginning of the AIS in most RGCs (20 ± 7.1 μm SD; Figures 1B,D). This highlights a significant difference to pyramidal neurons in V1, which in mice almost never showed a gap between proximal AIS and soma, but if a gap was detectable, the distance was significantly smaller (P28 visual cortex 2.3 ± 1.5 μm SD; Figures 1C,D).

To investigate the nanoscale architecture of the AIS in RGCs, we utilized STED microscopy and Single Molecule Localization Microscopy (SMLM). Analysis revealed that the ankG and βIV-spectrin scaffold is periodically spaced with distances of approximately 181 ± 26.08 nm SD between single fluorescent signal peaks (STED, Figures 2A–D; For SMLM: 205.6 nm ± 42.22 nm SD Supplementary Figures S2A,B,C). These findings confirm that RGC AIS are of similar nanostructure as other neuron populations, for which a scaffold with ∼190 nm spacing has been reported (D’Este et al., 2015; Leterrier et al., 2015). STED microscopy also revealed a striking deviation in AIS architecture compared to other neuron populations. RGC AIS often appear ‘looped’ or arranged in a corkscrew manner almost reminiscent of a double-helix structure (Figures 2A,B), with large ‘gaps’ between two single strands of axonal scaffold (Figure 2A).

### AIS of RGCs Undergo Dynamic Length Maturation During Postnatal Development

**FIGURE 3:**
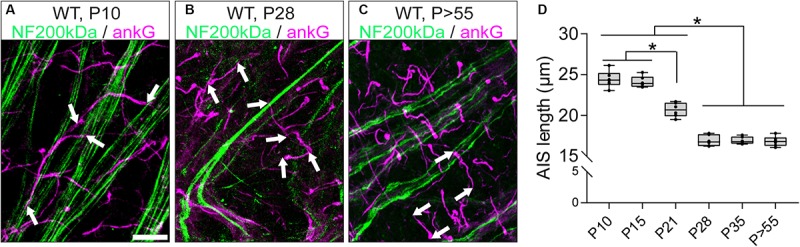
AIS length maturation during retinal development. **(A–C)** Whole mount retina stained against neurofilament 200 kDa (NF200, green) and the AIS scaffolding protein ankyrin-G (ankG, magenta) at P10 **(A)**, P28 **(B)**, and *P* > 55 **(C)** in wildtype mice. **(D)** Quantification of AIS length at various developmental stages, indicating longest AIS before eye-opening and length reduction throughout adulthood. One-way ANOVA. ^*^p ≤ 0.05. *n* = 6 animals (at least 100 AIS per animal). Scale bar = 10 μm.

The structural maturation of RGC AIS during the postnatal retinal development was investigated by immunofluorescence and confocal microscopy combined with AIS length analysis as previously published (Gutzmann et al., 2014; Schlüter et al., 2017). AIS length development was analyzed from P10 until adulthood (*P* > 55; Figure 3). AIS were the longest during the early postnatal period until eye-opening around P15 (P10: 24.45 ± 0.42 μm SD, P15: 24.11 ± 0.28 μm SD; Figures 3A,D). From P21 onward, AIS length significantly decreased, reaching stable length from P28 until adulthood (P21: 20.68 ± 0.36 μm SD, P28: 16.92 ± 0.28 μm SD, *P* > 55: 16.82 ± 0.25 μm SD; Figures 3B,D). In parallel, the cumulative length distribution of retinal AIS significantly shifted from a heterogeneous distribution to a homogenous length distribution at P21 (Supplementary Figures S3A–H). Across the three adult stages analyzed, RGC AIS length averaged at 16.9 μm ± 0.6 μm SD (P28, P35, and *P* > 55; Figure 3D), indicating that RGC AIS are shorter than AIS of other cell populations in adult wildtype mice, such as the visual cortex (average approximately 33 μm; Gutzmann et al., 2014), hippocampus (average approximately 29 μm; Kaphzan et al., 2011), or substantia nigra (average approximately 26 μm; Gonzalez-Cabrera et al., 2017).

### Synaptopodin Is Expressed in AIS of RGCs During Retinal Development

**FIGURE 4:**
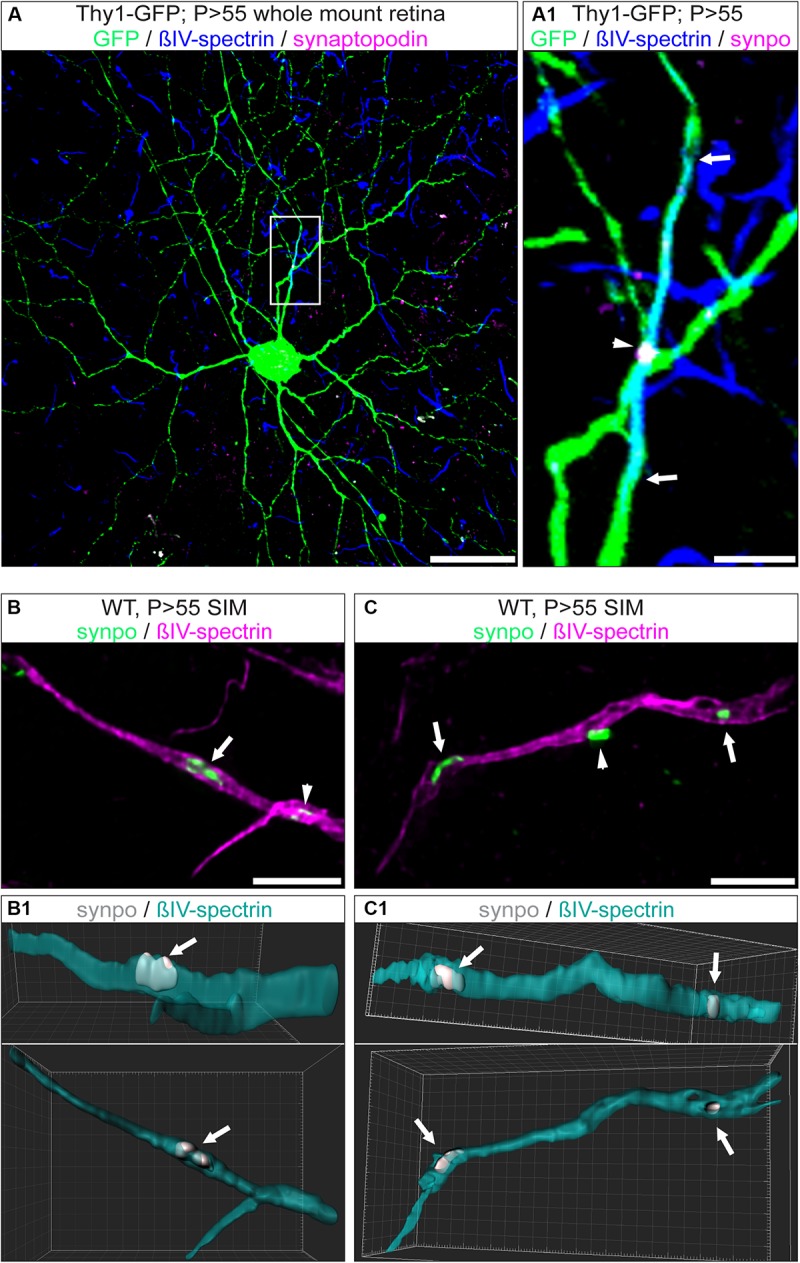
Expression of synpo in RGC AIS. **(A)** GFP-positive RGC (green) from a Thy1-EGFP animal stained against βIV-spectrin (blue) and synpo (magenta). **(A1)** Insert from boxed region in **(A)** with zoom-in view. The entire AIS length is outlined by arrows, the arrowhead highlights a single synpo cluster. **(B)** Higher resolution SIM image of selected AIS (magenta) with synpo clusters (green) and IMARIS reconstruction of this image **(B1)**. Only the cluster indicated by the arrow has been included in the reconstruction. Arrowhead indicates another synpo cluster (white) in the AIS (green). The three-dimensional expansion of the synpo cluster spanning the entire width of the AIS is visible in two different rotations (25° and 0° view from top). **(C)** SIM image of a single AIS, similar to image in **(B)**. Only synpo clusters indicated by arrows were reconstructed (**C1** with 25° and 0° rotation). Arrowhead indicates a synpo-positive structure (white) located outside the AIS (green). Scale bar **(A)** = 30 μm, **(B,C)** = 5 μm.

A subset of AIS of hippocampal and cortical neurons express the Ca^2+^-storing CO (Bas Orth et al., 2007; Sanchez-Ponce et al., 2012a; Schlüter et al., 2017). Considering the difference in morphology and function between RGCs and upstream neurons of the visual pathway, we asked whether RGC AIS also contain such intraaxonal Ca^2+^-stores. We applied immunofluorescence in retinal whole mounts to test whether synaptopodin (synpo), a core structural component of the CO, is expressed in RGC AIS (Figure 4). Interestingly, we found robust synpo expression in a subset of AIS of Thy1-positive RGCs and always confined to the borders of the AIS (Figures 4A–D).

A previous study using super resolution microscopy in cortical neurons proposed that synpo-positive COs are clustered at ankG-deficient sites in the AIS (King et al., 2014). We addressed the question whether these clusters appear at sites of ankG-deficiency in RGC AIS. Indeed, occasionally we found synpo clusters in the vicinity of gaps in the axonal scaffold (Figures 4B,C and Supplementary Figure S4B). However, by applying super resolution microscopy, we found that synpo-positive clusters are often located between rings of βIV-spectrin within the AIS (Supplementary Figure S4A). Occasionally, synpo clusters appeared as if they were located outside of the AIS (Figure 4C, arrowhead).

### The Size, but Not the Number of Synpo Clusters in AIS of RGCs Undergoes Changes During Retinal Development

**FIGURE 5:**
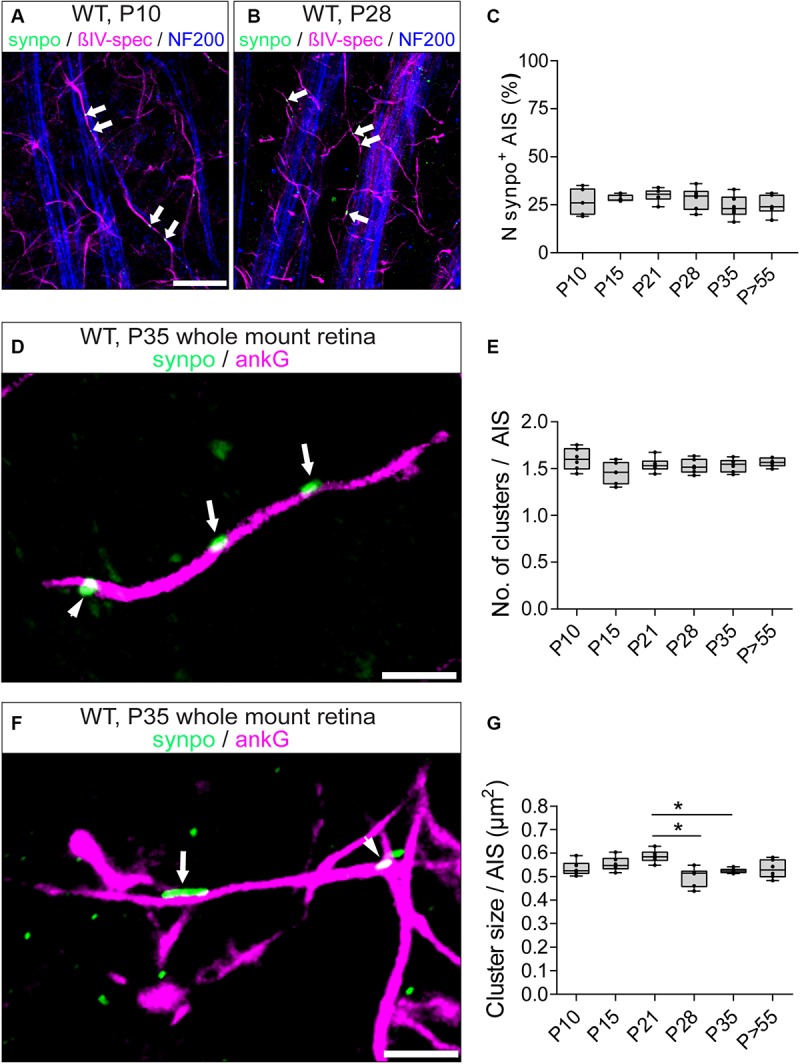
Synpo expression during development. **(A)** Whole mount retina from a P10 animal stained against βIV-spectrin (magenta), synpo (green) and NF200 (blue) in direct comparison to **(B)** from a P28 animal, showing that both contain approximately the same number of synpo^+^ AIS. Arrowheads highlight synpo clusters. **(C)** Quantification of the overall number of synpo^+^ RGC AIS during retinal development. **(D)** Representative example of several synpo clusters (green, arrows) in a single RGC AIS (ankG, magenta). Arrowhead indicates synpo-positive structure outside of the AIS. **(E)** Quantification of the number of synpo clusters in RGC AIS during retinal development, indicating stable number of clusters. **(F)** Representative example of an AIS with small (arrowhead) and large (arrow) synpo clusters. **(G)** Quantification of synpo cluster size in RGC AIS during retinal development, indicating a significant cluster size reduction after eye-opening. One-way ANOVA. ^*^p ≤ 0.05, *n* = 6 animals (at least 100 AIS per animal). Scale bar **(A,B)** = 20 μm, **(C,D)** = 5 μm.

In visual cortex neurons, synpo clusters undergo a dynamic regulation in both size and number during development (Schlüter et al., 2017). Here, we investigated synpo expression in RGC AIS during postnatal retinal development. Synpo expression in AIS was first observed at P10 in 26.5 ± 6.54% SD of RGC AIS (Figures 5A,C). During further postnatal development, the percentage of synpo-positive AIS in the retina remained stable and did not undergo any significant changes (i.e., P28: 28.17 ± 5.78% SD; Figures 5B,C). These data suggest that the presence of synpo in RGC AIS is not influenced by visual input mediated by the eye-opening phase around P13,14.

However, it remained an open question whether synpo clusters undergo similar dynamic changes during development as in V1 (Schlüter et al., 2017). Therefore, we examined the developmental changes of synpo expression in RGC AIS and quantified the number (Figures 5D,E) and size (Figures 5F,G) of synpo-positive clusters per AIS. At P10, the average number of synpo clusters per AIS was 1.6 ± 0.05 SD (Figure 5E). During further retinal development, the number of synpo clusters remained stable (i.e., P35: 1.53 ± 0.03 SD; Figure 5E), recapitulating the finding regarding the percentage of synpo-expressing AIS during retinal development (Figure 5C). The size of synpo-positive clusters in RGC AIS was 0.53 ± 0.01 μm^2^ SD at P10 and remained stable until P21 (0.59 ± 0.01 μm^2^ SD; Figure 5G). In comparison to the stable development of number of synpo-positive clusters per AIS, the size significantly decreased from P21 to P28 and P35, respectively (P28: 0.50 ± 0.02 μm^2^ SD, P35: 0.52 ± 0.004 μm^2^ SD; Figure 5G). During further postnatal development, synpo cluster sizes reached a plateau, which was maintained throughout adulthood (*P* > 55: 0.53 ± 0.02 μm^2^ SD; Figure 5G).

### Synpo Expression in RGC AIS Seems to Reduce and Stabilize AIS Length During Retinal Development

**FIGURE 6:**
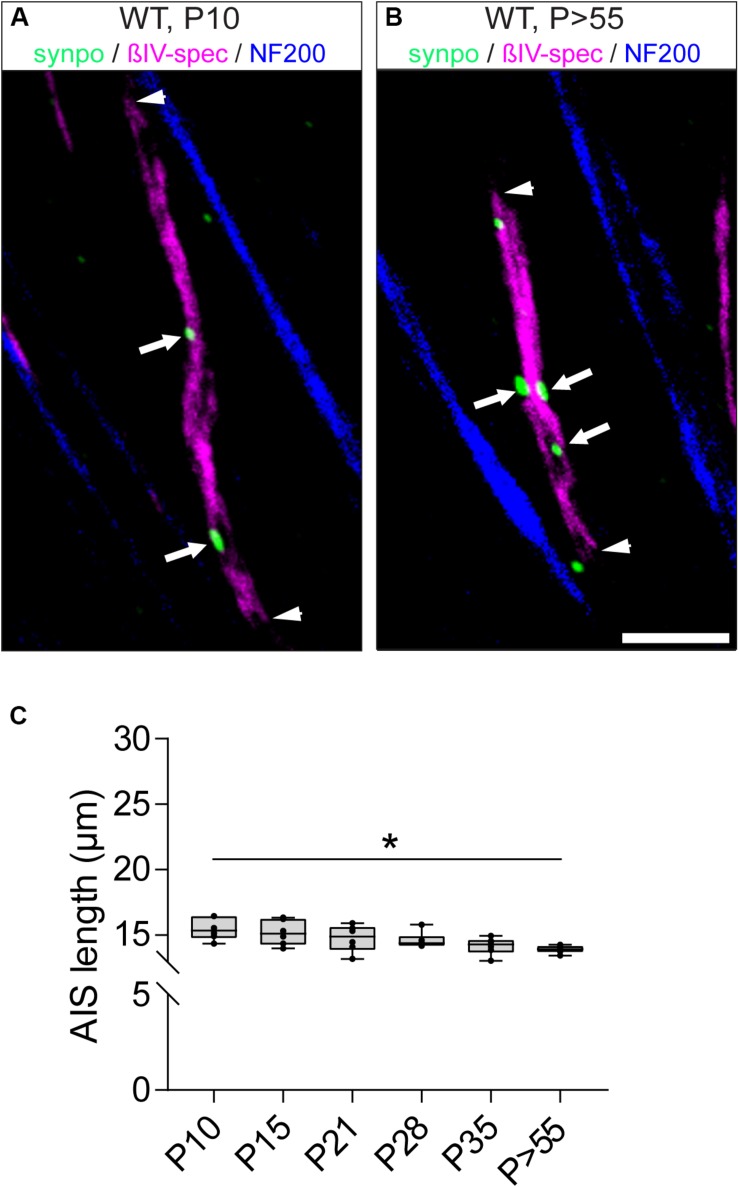
Length maturation of synpo^+^ RGC AIS during development. **(A)** Example of a P10 (long) synpo^+^ RGC AIS (βIV-spectrin, magenta and synpo, green). Arrowheads indicate entire AIS, arrows show synpo clusters within the AIS. **(B)** Example of a *P* > 55 (short) synpo^+^ RGC AIS (βIV-spectrin, magenta and synpo, green). Arrowheads indicate entire AIS, arrows show synpo clusters within the AIS. **(C)** Quantification of AIS length only in the subset of synpo^+^ RGC AIS during development, indicating a gradual length decrease. One-way ANOVA. ^*^p ≤ 0.05, *n* = 6 animals (at least 100 AIS per animal). Scale bar = 5 μm.

Since synpo-positive AIS display shorter AIS length and reduced dynamic length maturation during visual cortex development (Schlüter et al., 2017), we analyzed the developmental changes of length in the subset of synpo-expressing RGC AIS (Figure 6), and compared it to the length maturation of the entire AIS population in the retina (Figure 3). At P10, synpo-positive AIS have an average length of 15.48 ± 0.35 μm SD (Figures 6A,C). During further postnatal development, AIS length continuously decreased (i.e., P28: 14.60 ± 0.25 μm SD; Figures 6B,C) and reached a plateau in adulthood (*P* > 55: 13.91 ± 0.12 μm SD; Figure 6C), at which time AIS length is significantly reduced compared to the early postnatal period at P10. In parallel, the cumulative length distribution of RGC AIS was significantly homogeneous already early in development (Supplementary Figures S5A–H). Further, the average length of synpo-positive AIS was significantly shorter during retinal development as compared to the entire AIS population [Figure 6C, compare to Figure 3D; i.e., P21 (all AIS) vs. P21 (synpo^+^ AIS: 20.68 ± 0.88 μm SD vs. 14.75 ± 1.02 μm SD)]. These findings suggest that similar to the AIS of pyramidal neurons in the visual cortex (Schlüter et al., 2017), the presence of synpo-positive clusters within the AIS might reduce and possibly stabilize AIS length in RGCs during postnatal retinal development.

### Visual Deprivation During Retinal Development Impairs AIS Length Maturation in the Retina

**FIGURE 7:**
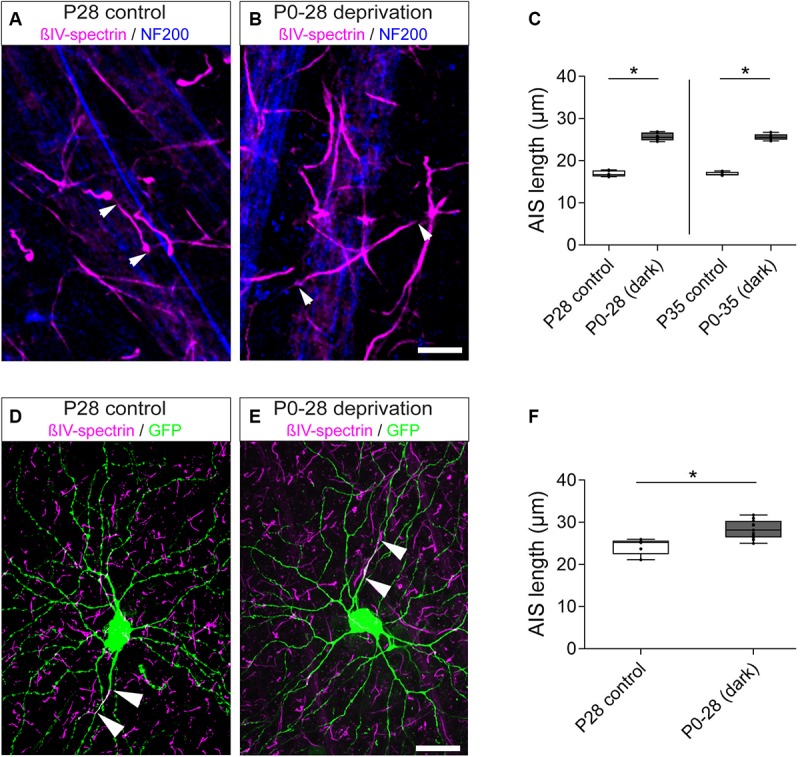
Length maturation of RGC AIS after sensory deprivation. **(A)** Example of P28 control RGC AIS (βIV-spectrin, magenta and NF200kDa, blue) in wildtype retina. Arrowheads indicate entire (short) AIS. **(B)** Example of visually deprived RGC AIS (animals reared in darkness from birth to P28; βIV-spectrin, magenta and NF200kDa, blue). Arrowheads indicate entire (long) AIS. **(C)** Quantification of AIS length in control (P28, P35) and visually deprived RGC AIS (P0–28, P0–35), indicating significant AIS length increase in both conditions. **(D)** Example of a control RGC from a Thy1-GFP animal with short AIS (arrowheads; βIV-spectrin, magenta and GFP, green). **(E)** Example of a visually deprived RGC from a Thy1-GFP animal with long AIS (arrowheads; βIV-spectrin, magenta and GFP, green). **(F)** Quantification of AIS length in Thy1-GFP animals specifically for the subset of RGC_A_ cells in control (*P* > 28) and visually deprived conditions (P0–28), indicating significant AIS length increase. Unpaired *t*-test. ^*^p ≤ 0.05, *n* = 6 animals (at least 100 AIS per animal). Scale bar **(A,B)** = 10 μm; **(D,E)** = 20 μm.

Recent studies have demonstrated that sensory deprivation prevents the structural maturation of the AIS in auditory, visual and somatosensory system neurons during development (Kuba et al., 2010; Gutzmann et al., 2014; Schlüter et al., 2017; Jamann and Engelhardt, unpublished). In fact, AIS maturation seems to be an activity-dependent process during which network activity contributes to the final mature length of AIS in cortical principal neurons (reviewed in Jamann et al., 2018). Considering the significant AIS length reduction we observed after eye-opening around P15 in RGC AIS (Figure 3), we hypothesized that a similar effect also contributes to AIS length maturation in the retina. To test this, we used visual deprivation protocols and reared mice in complete darkness from birth until P28 and P35, respectively. We measured AIS length in these animals and compared it to that of control animals kept under normal light/dark conditions (Figure 7). Similar to the observations during visual cortex development (Gutzmann et al., 2014), dark-rearing led to a significant increase in AIS length at P28 and P35 (P28 control vs. dark: 16.90 ± 0.28 μm SD vs. 25.69 ± 0.39 μm SD, Figures 7A–C; P35 control vs. dark: 16.92 ± 0.18 μm SD vs. 25.62 ± 0.31 μm SD, Figure 7C).

Of note, RGCs are heterogeneous regarding their morphology, firing properties and function. We therefore used data obtained from Thy1-GFP mice to further classify the different RGCs in our dataset as outlined in the methods. We found that most Thy1-positive RGCs belong to class A1 and A2, whereas other classes were represented only by up to ca. 10% of cells (Supplementary Figure S1C). Due to the low number of RGC_B_, RGC_C_, and RGC_D_ cells, sufficient detection of reliable numbers of cells and related AIS in these classes was not pursued.

Selecting only RGC_A_ neurons for analysis, we found similar AIS elongation after dark rearing in this subtype [Figures 7D–F; control (*P* > 28) vs. dark (P28): 24.29 ± 1.95 μm SD vs. 28.33 ± 2.22 μm SD]. In these same cells, no significant difference in the gap between proximal AIS and soma was observed [Supplementary Table 2; control (*P* > 28) vs. dark (P28): 22.70 ± 3.44 μm SD vs. 22.80 ± 4.06 μm SD]. Size frequency histograms of AIS length in all RGC classes further highlighted that dark-rearing until P28 and P35 led to an AIS length distribution similar to that found in young animals, suggesting that neurons maintain a juvenile AIS length (Supplementary Figures S3A–H). Thus, visual deprivation seems to prevent the structural maturation and developmental shortening of AIS length in RGCs during postnatal periods.

### Visual Deprivation During Retinal Development Increases Synpo Expression and Length of Synpo-Positive RGC AIS

**FIGURE 8:**
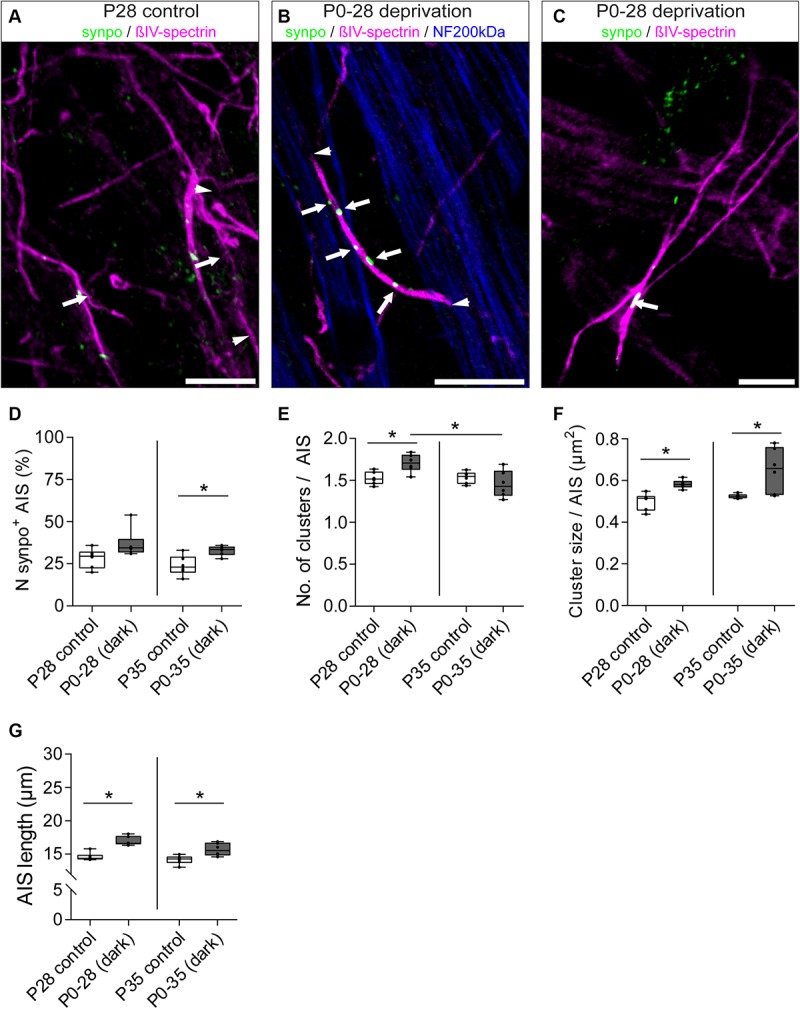
Dynamic remodeling of synpo cluster number and size after sensory deprivation. **(A)** Example of P28 control RGC AIS (βIV-spectrin, magenta and synpo, green). Arrowheads indicate entire AIS; arrows highlight single synpo clusters. **(B)** Example of RGC AIS in P28 dark-reared mice showing increased number of clusters (arrows) and AIS length (arrowheads); same in **(C)** showing increased synpo cluster size (arrows). **(D)** Quantification of number of synpo^+^ RGC AIS, indicating an increase in synpo clusters in AIS after 35 days of deprivation. **(E)** Quantification of number of synpo clusters, indicating a significant increase in synpo clusters after 28, but not 35 days of sensory deprivation. **(F)** Quantification of synpo cluster size showing significant increase both after 28 and 35 days of sensory deprivation. **(G)** Quantification of overall AIS length after sensory deprivation, showing significant length increase in both conditions. Unpaired *t*-test and one-way ANOVA. ^*^p ≤ 0.05, *n* = 6 animals (at least 100 AIS per animal). Scale bar **(A,B)** = 10 μm, **(C)** = 5 μm.

We next hypothesized that visual input influences not only AIS length maturation, but also the development of synpo clusters in AIS of RGCs. Thus, we compared changes in synpo protein expression in RGC AIS in control animals with animals subjected to dark-rearing for 28 and 35 days (Figures 8A–G). We quantified the percentage of synpo-expressing AIS as well as the number and size of synpo-positive clusters per AIS. Regarding the percentage of synpo-expressing AIS, we found that the subset of AIS that contain synpo-positive clusters increased after dark-rearing for 28 and 35 days, which was significant in P35 animals (P28 control vs. dark: 28.17 ± 2.36% SD vs. 36.83 ± 3.50% SD, Figure 8D; P35 control vs. dark: 24.0 ± 2.41% SD vs. 32.8 ± 1.2% SD; Figure 8D). Further, dark-rearing for 28 days resulted in a significant increase in the average number of synpo clusters per AIS (P28 control vs. dark: 1.53 ± 0.03 SD vs. 1.71 ± 0.04 SD; Figures 8A,B,E) as well as significant increase of synpo cluster size per AIS (P28 control vs. dark: 0.50 ± 0.02 μm^2^ SD vs. 0.58 ± 0.01 μm^2^ SD; Figures 8A,C,F). Longer periods of visual deprivation until P35 resulted in an unchanged number of synpo-positive clusters per AIS as compared to the control conditions (P35 control vs. dark: 1.53 ± 0.03 SD vs. 1.46 ± 0.07 SD; Figure 8E). Instead, an increase of the size of synpo-positive clusters in AIS of P35 dark-reared mice was found when comparing them to P35 control mice and visually deprived P28 mice, respectively (P35 control vs. dark: 0.53 ± 0.004 μm^2^ SD vs. 0.65 ± 0.04 μm^2^ SD, Figure 8F; P28 dark: 0.58 ± 0.01 μm^2^; Figure 8F). These data suggest that visual input can influence number and size of synpo-positive clusters in AIS of RGCs during postnatal development. Moreover, synpo clusters might fuse within the AIS by further increasing in size when visual input is lacking for periods longer than P28 days. Interestingly, sensory deprivation led to an increase in RGCs that express synpo in their AIS; in other words, RGCs that normally would not express synpo, seemed to initiate expression after the lack of visual input during postnatal development.

Our findings indicate a potential AIS length-stabilizing effect of synpo in RGCs during postnatal development (Figure 6). Consequently, we questioned whether synpo-positive AIS still retained their ability to adapt their length to changes in visual input as seen for the entire retinal AIS population (Figure 7). Therefore, we quantified the length of AIS in the subset of synpo-expressing RGCs in mice that were dark-reared for 28 and 35 days and compared them to control mice raised under normal light/dark conditions (Figure 8G). Under all dark-rearing conditions, a significant increase in the length of synpo-positive AIS was observed (P28 control vs. dark: 14.60 ± 0.25 μm SD vs. 16.96 ± 0.28 μm SD, Figure 8G; P35 control vs. dark: 14.16 ± 0.27 μm SD vs. 15.68 ± 0.40 μm SD; Figure 8G). Further, length distribution histograms of synpo-positive AIS highlighted that visual deprivation led to a significant shift from homogenous to heterogeneous length distributions (Supplementary Figures 5A–H). In conclusion, even though the presence of synpo in AIS of RGCs seems to stabilize AIS length during postnatal development under physiological conditions, synpo-positive AIS maintain the ability to dynamically adjustment their length after loss of visual input.

## Discussion

In V1, where visual input is processed, the AIS of pyramidal neurons is highly plastic during development and after sensory deprivation (Gutzmann et al., 2014). For RGCs, which constitute a downstream neuron population in the visual pathway, the precise morphology as well as putative plasticity of AIS has not been studied in detail so far. In the present work, we investigated the structural maturation of AIS and the CO in RGCs during retinal development and after sensory deprivation. Our work highlights that (1) RGC AIS have a periodically spaced scaffold of ankG/βIV-spectrin rings; (2) AIS length in RGCs changes with the maturation of the retina during postnatal development; (3) RGC AIS express the specific CO marker synpo, which is located within the ankG/βIV-spectrin scaffold occasionally in close proximity to ankG/βIV-spectrin deficient sites; (4) the percentage of synpo-positive AIS is stable during development, but synpo cluster sizes change with AIS maturation; and (5) visual deprivation prevents the maturation of AIS shortening in RGCs and increases synpo expression in AIS during postnatal development.

Taken together, our data indicate that the presence of synpo could provide structural stability to RGC AIS during periods of refinement of retinal circuits. Further, loss of visually driven synaptic input prevents the structural maturation of AIS length shortening as well as increases synpo expression in RGCs.

### AIS of RGCs Have Similar Architectural Features as Principal Cortical Neurons

Here, we analyzed AIS positions along the axon by measuring the distance of the AIS to the RGC soma in adult animals. We found that RGC AIS appear with a significant distance to the soma compared to pyramidal neurons in V1. AIS length and/or AIS position along the axon is proposed to impact neuronal excitability depending largely on the corresponding somatodendritic morphology of neurons (Gulledge and Bravo, 2016; Hamada et al., 2016; Kole and Brette, 2018). Thus, it is likely that the observed diversity in AIS position along the axon of RGCs in our study is linked to the variability of somatodendritic morphology within the different rodent RGC classes (Sun et al., 2002a,b; Sanes and Masland, 2015). As a result, optimal neuronal excitability could be adjusted by changing AIS length/position to individually limit or promote action potential generation.

By applying super resolution microscopy in neurons *in vitro*, the robust nanoscale organization along the AIS submembrane scaffold has been identified as a ∼190 nm periodic ring-like architecture formed by submembrane actin bands connected by longitudinal head-to-head βIV-spectrin subunits, which extend throughout the axon (Xu et al., 2013; D’Este et al., 2015; Leterrier et al., 2015). Despite the diverse AIS morphology in RGCs, our super resolution imaging data revealed a consistent nanoscale architecture along the AIS of RGCs. As seen in neurons *in vitro*, we observed a periodic arrangement of βIV-spectrin with distances of ∼180 nm in RGC AIS. Within the AIS, we identified synpo-positive structures, corresponding to COs, localized underneath the membrane scaffold. Recently, it has been speculated that synpo-positive clusters within the AIS of cortical neurons *in vitro* and *in vivo* are localized at gaps in the AIS scaffold, which are deficient of ankyrin-G expression (King et al., 2014). These ankG and βIV-spectrin deficient sites are apparent in images acquired by super resolution imaging (STORM and STED) in AIS of neurons *in vitro* (D’Este et al., 2015; Leterrier et al., 2015). By applying SIM and SMLM, we observed such gaps in the RGC AIS scaffold, which were deficient of βIV-spectrin or ankG expression. However, these gaps were only occasionally filled with synpo-positive clusters and these clusters were often detected at sites in the AIS where no obvious gaps were visible. It is important to keep in mind that the super resolution methods applied here and in other studies have limitations. For example when using SMLM, the lack of three-dimensional information might impact the interpretation of data. Moreover, a high density fluorescence labeling is required for SMLM to allow detection of positions of individual molecules for reconstructing a high resolution image (Klein et al., 2014). This could also impact the accuracy of measurements of positions of single molecules as we observed for measurements of distances between single ankG/βIV-spectrin signals (∼180 nm by STED vs. ∼200 nm by SMLM). A combination of methods should help to address the still unresolved question about ‘gaps’ in the AIS scaffold and possible colocalization of synpo clusters in these domains. Intriguingly, King and others speculated that gaps in the ankG/βIV-spectrin-actin scaffold are required to accommodate high density protein complexes that might be crucial for the assembly and functioning of axo-axonic GABAergic synapses at the AIS (Kosaka, 1980; Benedeczky et al., 1994; King et al., 2014). So far, the presence of such GABAergic synapses at RGC AIS is supported by an early electron microscopy study of the macaque monkey retina (Koontz, 1993). However, work supporting these findings in rodent RGCs is currently lacking. Furthermore, occasionally we observed synpo clusters that appeared as if they were partially located outside of the AIS. This phenomenon could have a technical explanation. Considering that ankG immunoreaction does not highlight the outside of the axonal membrane, but rather the underlying scaffold of the axon and hence, spans less axonal diameter, the actual complete axonal diameter may be underestimated. In addition, it is intriguing to speculate that the observation is further indication of a synpo/CO-subdomain in the AIS. There is evidence from hippocampal pyramidal neuron AIS that the synpo-positive clusters may represent ‘spine-like’ protrusions in the vicinity of – or as a direct contact site for – GABAergic synapses (Somogyi et al., 1983).

### Length of RGC AIS Correlates With Retinal Maturation During Postnatal Development

The AIS of RGCs is located proximally in the unmyelinated part of the axon and therefore, is separated spatially from the distal myelinated part (Hildebrand and Waxman, 1983; Koontz, 1993). The molecular composition of RGC AIS is comparable to that of other neurons across different species, and has been demonstrated by several studies (Hildebrand and Waxman, 1983; Carras et al., 1992; Koontz, 1993; Boiko et al., 2003; Van Wart et al., 2007; Zhang et al., 2015). Yet, the developmental maturation of AIS length in retinal neurons remained unexplored.

AIS length and location has been linked to the excitability of neurons (Grubb and Burrone, 2010; Kuba et al., 2010; Hamada et al., 2016; Jamann et al., 2018). Data support the hypothesis that longer AIS facilitate action potential generation and thus contribute to increased neuronal excitability. Interestingly, neuronal activity and excitability are inversely related, i.e., higher activity results in AIS shortening and lower activity leads to AIS elongation, a mechanism reminiscent of homeostatic plasticity (reviewed in Adachi et al., 2015; Wefelmeyer et al., 2016).

During cortical development, AIS undergo structural length maturation from increased AIS length at embryonic and early postnatal ages to a shortening at later postnatal stages and in adulthood (Cruz et al., 2009; Galiano et al., 2012; Fish et al., 2013; Gutzmann et al., 2014; Jamann et al., 2018). Here, we addressed the question whether similar developmental profiles apply for RGCs. Indeed, at postnatal ages around eye-opening (P10/15), AIS were longest with heterogeneous length distributions indicative of ‘juvenile’ AIS in V1 (Gutzmann et al., 2014). A characteristic feature of the developing retina is spontaneous periodic activity (reviewed in Firth et al., 2005). Spontaneous retinal waves emerge around E16.5, undergo different stages, and last until eye-opening (reviewed in Huberman et al., 2008). Vision in mice begins around P11 through naturally closed eye lids (Krug et al., 2001). The simultaneous presence of both spontaneous retinal activity and visually driven activity might provoke the increase in AIS length of RGCs in young animals. In turn, increased excitability is proposed to be acquired for early development in sensory cortices (Oswald and Reyes, 2008; Frangeul et al., 2017). It is conceivable that increased AIS length during retinal development leads to increased RGC excitability, which contributes to the refinement of axonal projections from the retina to the visual centers of the brain. Indeed, glutamate release from bipolar cells during spontaneous retinal waves from P10–12 regulates circuit development in the retina, and patterns of RGC activity propagating forward shape the wiring of circuits in the lateral geniculate nucleus, superior colliculus and in V1 (reviewed in Kerschensteiner, 2016). Interestingly, RGC activity is fundamental not only for proper development. Abnormalities in RGC activity were observed during development of pathologies and increased activity is discussed to decelerate the degenerative progression of retinal disease (Risner et al., 2018).

Around eye-opening (P13,14), spontaneous retinal activity begins to disappear and is finally absent around P21 (Demas et al., 2003). The complete replacement of spontaneous activity with visually driven activity possibly triggered the decrease in AIS length in RGCs at P21, accompanied by a parallel homogenous AIS length distribution, which is characteristic for mature AIS in adult animals (Gutzmann et al., 2014). With proceeding development, AIS of RGCs decreased further in length, underlined by progressively more homogeneous AIS length distributions. Our findings indicate that AIS begin to mature when spontaneous retinal activity ends, suggesting that visual experience is important for the maturation of RGC AIS.

### Synpo Expression in RGC AIS Changes During Retinal Development and Stabilizes AIS Length Maturation

In rodent V1, synpo expression in the AIS begins postnatally and is most prominent in structurally mature AIS of pyramidal neurons during early critical periods of cortical development (Schlüter et al., 2017). In the present study, the first synpo-positive clusters appeared in RGC AIS in P10 mice. Of note, maturation changed the size of synpo-positive clusters in RGC AIS, but the number of clusters per AIS was unaltered. In comparison to our study in the developing V1 (Schlüter et al., 2017), synpo expression was more constant during retinal development. The subset of AIS, which express synpo, amounted to ∼24 – 28% between the different ages and taking all RGC types into account. It should be pointed out that synpo clusters may possibly be associated with specific RGC classes, however, due to the overall scarcity of RGC_A_ cells in our study, we cannot provide more detailed data at this point. The question whether synpo/CO segregates into specific RGC classes will have to be addressed in future studies. Interestingly, synpo-positive clusters within AIS were significantly different in size between P21 and P28 as well as P21 and P35. What could trigger the size reduction of synpo clusters during late postnatal development? One of the most important messengers for the induction of neuronal signaling during retinal development is Ca^2+^ (Firth et al., 2005; Kerschensteiner, 2016). The CO and synpo have been implied in regulating local AIS Ca^2+^ trafficking (Benedeczky et al., 1994; Sanchez-Ponce et al., 2012b; King et al., 2014). Ca^2+^ sensitive channels (Inositol 1,4,5-trisphosphate receptors, ryanodine receptors) and Ca^2+^ pumps (i.e., sarco/endoplasmic reticulum Ca^2+^-ATPase) associated with the CO are discussed to boost local cytosolic Ca^2+^ transients during action potential firing through Ca^2+^-induced Ca^2+^ release from internal Ca^2+^ stores (Berridge, 1998; Segal, 2018). This Ca^2+^ signaling amplification in turn could impact axonal membrane potential properties and neuronal excitability (Berridge, 1998; Segal, 2018), and thus AIS length.

In the developing retina, RGCs spontaneously fire periodic bursts of action potentials with accompanying large increases of intracellular Ca^2+^ levels (reviewed in Firth et al., 2005). In mice, stage III – glutamatergic activity emerges from P10-14 (reviewed in Huberman et al., 2008; Kerschensteiner, 2016), declines around the time of eye opening as light-evoked signals begin to drive retinal activity, and are finally absent around P21 (Demas et al., 2003). The initial expression of synpo in RGC AIS at P10 may be linked to the appearance of stage III retinal glutamatergic waves and the imminent onset of vision. Early visual, experience along with the replacement of glutamatergic waves with light-evoked, signals may trigger a later decrease of sizes of synpo-positive clusters along with AIS length maturation of RGCs. Further, the density of synapses in the outer and IPL peaks around P21 (Xu and Tian, 2004). From P22 until P27, an increase of spontaneous excitatory and inhibitory postsynaptic currents emerges, which enhances RGC synaptic input more than fourfold (Tian and Copenhagen, 2001). This increase of synaptic input may change the firing properties of RGCs, which in turn might lead to the observed remodeling of synpo and the CO in retinal AIS.

In parallel with the emergence of synpo clusters in RGC AIS at P10, we observed a homogenous AIS length distribution compared to the entire AIS population in the retina at this age reflecting mature AIS (Gutzmann et al., 2014; Schlüter et al., 2017). The maturation of synpo-positive AIS early in development suggests that synpo presence in the AIS is a sign of structural maturity. Similar findings have been described for synpo expression in the AIS of pyramidal neurons during V1 development (Schlüter et al., 2017) or in dendritic spines (Mundel et al., 1997; Czarnecki et al., 2005).

### Dark-Rearing Prevents Structural AIS Maturation and Remodels COs Within RGC AIS

Of note, the subset of RGCs that express synpo in their AIS, whose percentage was small throughout retinal development, were ∼120% higher in dark-reared animals. In all deprivation conditions, synpo-positive AIS were ∼110% longer than AIS in control animals of the same age and reached AIS length comparable to those observed in younger control animals. Further, sizes of synpo-positive clusters within RGC AIS were increased after dark-rearing. This could indicate that existing synpo-positive clusters fuse within the AIS after loss of visually driven synaptic input. The expansion of the ER most likely regulates the capacity of internal Ca^2+^ stores (Sammels et al., 2010). Likewise, the formation of increased synpo-positive clusters is proposed to enhance Ca^2+^ storage capacity in the spine apparatus in dendrites (Vlachos et al., 2013; Verbich et al., 2016) as well as in the CO in AIS (Schlüter et al., 2017) as a homeostatic response to loss of synaptic input. We speculate that both elongated AIS and expanded synpo-positive clusters within the AIS increase the excitability of RGCs as well as Ca^2+^ currents in their AIS to compensate for the visual deprivation-triggered reduction in retinal network activity.

Finally, considering the significant heterogeneity of RGC classes, we would like to point out that further classification of RGC_A_ into ON and OFF cells might be of interest since dark rearing might have differential effects on ON and OFF cells; it should silence ON cells and activate OFF cells, with presumably opposite AIS plasticity. Future studies in animal models more suited to discern a larger number of RGC classes will be able to shed light on this interesting question.

## Conclusion

Our findings suggest that changes in synpo expression are linked to different stages of activity-driven processes in the developing retina. Moreover, we identified a possible role for both AIS and synpo/CO plasticity during homeostatic responses of visual input-deprived RGCs to reduced retinal network activity. Future studies could therefore focus on the subtypes of synpo-expressing RGCs, their intrinsic AIS plasticity and how it pertains to the development and maturation of functional visual circuits.

## Data Availability

The datasets generated for this study are available on request to the corresponding author.

## Ethics Statement

All animal protocols were approved by the Medical Faculty Mannheim Animal Research Board, Heidelberg University, as well as the State of Baden-Württemberg, Germany, and were conducted in accordance with Heidelberg University Guidelines on the Care of Laboratory Animals.

## Author Contributions

AS, SR, and ME conceptualized and designed the study. AS, SR, DD, JMJ, SV, and JH contributed to data acquisition and programming. AS, SR, DD, JMJ, and ME analyzed the data. AS, SR, CS, and ME interpreted the data. AS, DM, and ME prepared the manuscript. All authors approved the final version to be published.

## Conflict of Interest Statement

SR joined DELMIC B.V. in the Netherlands, a company producing solutions for correlative light and electron microscopy, after completion of all work relating to the current study. Therefore SR declares no conflict of interest. JH works for Abberior Instruments GmbH, a company producing STED microscopes. DM is cofounder and shareholder of FundaMental Pharma GmbH, which has no conflict of interest with the present work. The remaining authors declare that the research was conducted in the absence of any commercial or financial relationships that could be construed as a potential conflict of interest.
