# Supplementary material for: Dynamic Regulation of Synaptopodin and the Axon Initial Segment in Retinal Ganglion Cells During Postnatal Development
Source: Front Cell Neurosci. 2019 Jul 30;13:318. doi: 10.3389/fncel.2019.00318 (PMC6682679; doi:10.3389/fncel.2019.00318)
Supplement: Supplementary file 1 [file Data_Sheet_1.pdf]

Supplementary Material

Dynamic regulation of synaptopodin and the axon initial segment in retinal ganglion cells during postnatal development

Annabelle Schlüter[1,2,] Sabrina Rossberger[3#], Dominik Dannehl[1], Jan Maximilian Janssen[1], Silke Vorwald[1], Janina Hanne[4], Christian Schultz[1], Daniela Mauceri[2], Maren Engelhardt[1]*

[1] Institute of Neuroanatomy, Center for Biomedical Research and Medical Technology (CBTM), Medical Faculty Mannheim, Heidelberg University, Germany

[2] Neurobiology, Interdisciplinary Center for Neurosciences, Heidelberg University, Heidelberg, Germany

[3] Kirchhoff Institute of Physics, Applied Optics, Heidelberg University, Germany

[4] Abberior Instruments GmbH, Heidelberg, Germany

[#] Current address: DELMIC B.V., Kanaalweg 4, 2628 EB Delft, The Netherlands

***Correspondence:**

Dr. Maren Engelhardt
Institute of Neuroanatomy
Center for Biomedical Research and Medical Technology (CBTM)
Medical Faculty Mannheim, Heidelberg University
Ludolf-Krehl-Str. 13-17
68167 Mannheim
Germany
Phone: +49-621-38371556
Email: maren.engelhardt@medma.uni-heidelberg.de

Supplementary Table 1 Specification of antibodies with indication of catalog number, working dilution, previously conducted controls, sources and references where available. *KO* absence of immunostainings in knock out animals, *IHC* immunohistochemistry, *IP* immunoprecipitation, *WB* western blot, *rb* rabbit, *ms* mouse, *ch* chicken, *gp* guinea pig

**Antibody**<br>**Clone/type; Catalog Number**	**Dilution in IF**	**Reported specificity**				**Source**<br>**Reference**
		**KO**	**IHC**	**IP**	**WB**	
Synaptopodin (gp)<br>163 004	1:500	X	X		X	Synaptic Systems GmbH, Göttingen, Germany<br>(Schlüter et al., 2017)
Synaptopodin (ms)<br>G1D4 ; BM5086P	1:100	X	X		X	Acris Antibodies GmbH, Herford, Germany<br>(King et al., 2014)
Ankyrin-G (ms)<br>N106/36; 73-146	1:250	X	X		X	UC Davis/NIH NeuroMab Facility, CA, USA<br>(Engelhardt et al., 2013)
ßIV-spectrin (rb)<br>amino acids 2237-2256 of human ßIV-spectrin	1:500	X	X		X	Selfmade<br>(Gutzmann et al., 2014)
200kDa Neurofilament (ch)<br>pAb IgY<br>ab72996	1:1000		X		X	Abcam Cambridge, UK<br>(Engelhardt et al., 2013)
GFP (ms)<br>3E6<br>11120	1:500		X	X	X	Thermo Fischer Scientific, Waltham, Massacchusetts, USA
NeuN (ms)<br>A60<br>MAB377	1:250		X		X	Millipore, Temecula, USA<br>(Gutzmann et al., 2014)
Goat anti-Mouse IgG (H+L)<br>Alexa Fluor 488<br>A-11001	1:1000					Thermo Fischer Scientific, Waltham, Massacchusetts, USA
Goat anti-Guinea Pig IgG (H+L) Alexa Fluor 488<br>A-11073	1:1000					Thermo Fischer Scientific, Waltham, Massacchusetts, USA
Goat anti-Mouse IgG (H+L)<br>Alexa Fluor 568; A-11004	1:1000					Thermo Fischer Scientific, Waltham, Massacchusetts, USA
Goat anti-Rabbit IgG (H+L)<br>Alexa Fluor 568; A-11011	1:1000					Thermo Fischer Scientific, Waltham, Massacchusetts, USA
Goat anti-Chicken IgG (H+L)<br>Alexa Fluor 647; A-21449	1:1000					Thermo Fischer Scientific, Waltham, Massacchusetts, USA
STAR 580<br>ST580	1:500					Abberior, Göttingen, Germany<br>(D'Este et al., 2016)
STAR 635P<br>ST635P	1:100					Abberior, Göttingen, Germany<br>(Schedin-Weiss et al., 2017)

Supplementary Table 2. Measured values for AIS length, AIS percentage, number and size of synpo clusters as well as AIS distance to soma with indication of age of animal, mouse strain and period of visual deprivation. Results indicate mean values and S.D. between the measured mean values for each age/condition. N is animal/age or conditions, with at least 100 AIS analyzed per animal.

	**age/condition**	**wildtype (control)**	**wildtype (visual deprivation)**
**AIS length (all) in µm** n=6	P10	24.45 ± 1.02	-
	P15	24.11 ± 0.69	-
	P21	20.68 ± 0.88	-
	P28	16.90 ± 0.68	25.69 ± 0.95
	P35	16.92 ± 0.45	25.62 ± 0.77
	P>55	16.82 ± 0.62	-
**AIS percentage ($synpo^+$) in %** n=6	P10	26.5± 6.54	-
	P15	28.33 ± 1.75	-
	P21	30.00 ± 3.52	-
	P28	28.17 ± 5.78	36.83 ± 5.78
	P35	24.00 ± 5.90	32.83 ± 2.93
	P>55	24.83 ± 5.12	-
**Synpo cluster number** n=6	P10	1.60 ± 0.12	-
	P15	1.45 ± 0.12	-
	P21	1.54 ± 0.08	-
	P28	1.53 ± 0.08	1.71 ± 0.11
	P35	1.53 ± 0.07	1.46 ± 0.16
	P>55	1.57 ± 0.05	-
**Synpo cluster size in $µm^2$** n=6	P10	0.53 ± 0.03	-
	P15	0.55 ± 0.03	-
	P21	0.59 ± 0.03	-
	P28	0.50 ± 0.04	0.58 ± 0.02
	P35	0.53 ± 0.01	0.65 ± 0.11
	P>55	0.53 ± 0.02	-
**AIS length ($synpo^+$) in µm** n=6	P10	15.48 ± 0.85	-
	P15	15.18 ± 0.96	-
	P21	14.75 ± 1.02	-
	P28	14.60 ± 0.62	16.96 ± 0.69
	P35	14.16 ± 0.66	15.68 ± 0.98
	P>55	13.91 ± 0.29	-

	**age**	**wildtype** V1	**Thy1-GFP** retina
**AIS distance to the soma in µm** n=5	P>55	2.26 ± 1.12	20.02 ± 7.09

	age	Thy1-GFP (control)	Thy1-GFP (visual deprivation)
**AIS length (RGC_A) in µm** n=5 (control) n=11 (visual deprivation)	P≥28	24.29 ± 1.95	28.33 ± 2.22
**AIS distance to the soma (RGC_A) in µm** n=5 (control) n=11 (visual deprivation)	P≥28	22.70 ± 3.44	22.80 ± 4.06

Supplementary Methods

Image acquisition in super resolution mode

SIM-images were recorded by illuminating the sample with an adjustable sinusoidal grating (period: 350 nm). The grating was shifted twice each time by 1/3 of the period into the direction of modulation (perpendicular to the grating stripes). This resulted in an automatic recording of three images of conventional resolution with different positions of the illumination grating. The sum of the three images with different phase positions corresponds to one image with homogenous widefield illumination. Afterwards, the grating was automatically rotated twice to 60° and -60° (total of three grating orientations). Again, three images of changing phase were recorded at each grating position. This finally resulted in a total of nine images (three phases x three orientation) to achieve a lateral isotropic resolution improvement. The two color channels were recorded sequentially (647 nm followed by 568 nm to avoid bleaching). The fluorescent signal for each image was integrated over 50 msec. Focal plane illumination intensities were approximately (46.8 ± 2.7) Wcm^2. For 3D-SIM-imaging, a total of nine images per z-layer were recorded. The distance between two images within the 3D-image stack was 200 nm.

SMLM images were recorded by illuminating the sample with a homogenous widefield illumination. The two color channels were imaged consecutively. A 2D time series for the color channel of the longer 671 nm wavelength was imaged, followed by a 2D image stack for the shorter 568 nm channel. Focal plane laser intensities were approximately (3.84 ± 0.1) $kWcm^2$, which was about two orders higher compared to SIM-imaging. The fluorescent signal was also integrated for 50 msec for each single image. An image series for one reconstructed 2D super resolution image typically consisted of 1000 images.

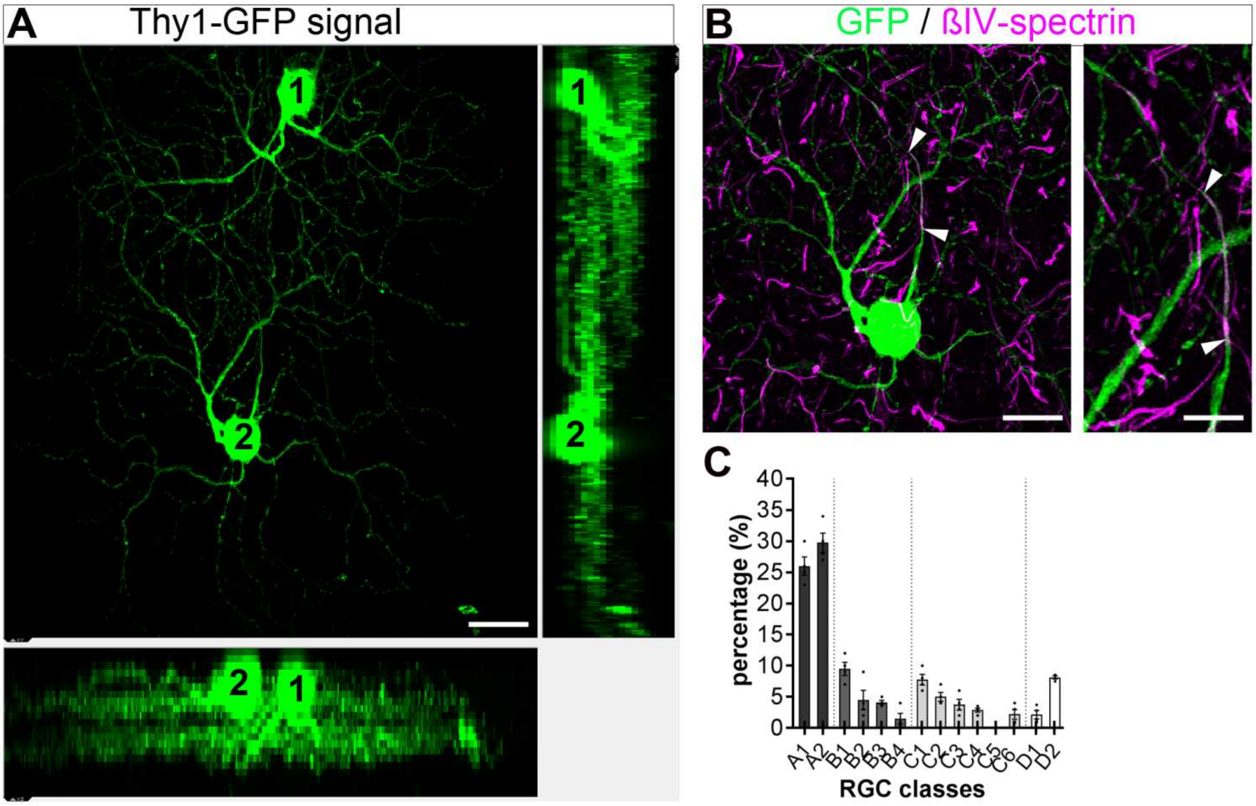

Supplementary Figure 1. Classification of RGCs in Thy1-GFP mice retinae. (A) Identification of OFF (cell 1) and ON (cell 2) retinal ganglion cells in one region of interest, highlighting how classification of RGCs into ON- and OFF-ganglion cells and colocalization of AIS markers with GFP-positive axons was achieved. XZY panels below and on right side of main panel show stratification of dendrites of these particular RGCs into different sublaminae of the inner plexiform layer. (B) Magnification of cell 2 from (A), now with AIS staining (βIV-spectrin, magenta) and arrowheads delineating the entire length of the AIS. (C) Quantification of percentage of RGCs belonging to different RGC classes (A1-A2, B1-B4, C1-C6, D1-D2). RGCs in Thy1-GFP mice were classified based on their soma size and dendritic tree diameter according to (O'Brien et al., 2014; Sun et al., 2002). Percentage of the different RGC classes was calculated in relation to the number of total GFP-positive RGCs in each retina (n=2 animals, 4 retinae total). Scale bars A = 30 µm; B = 20 µm; B side panel = 10 µm.

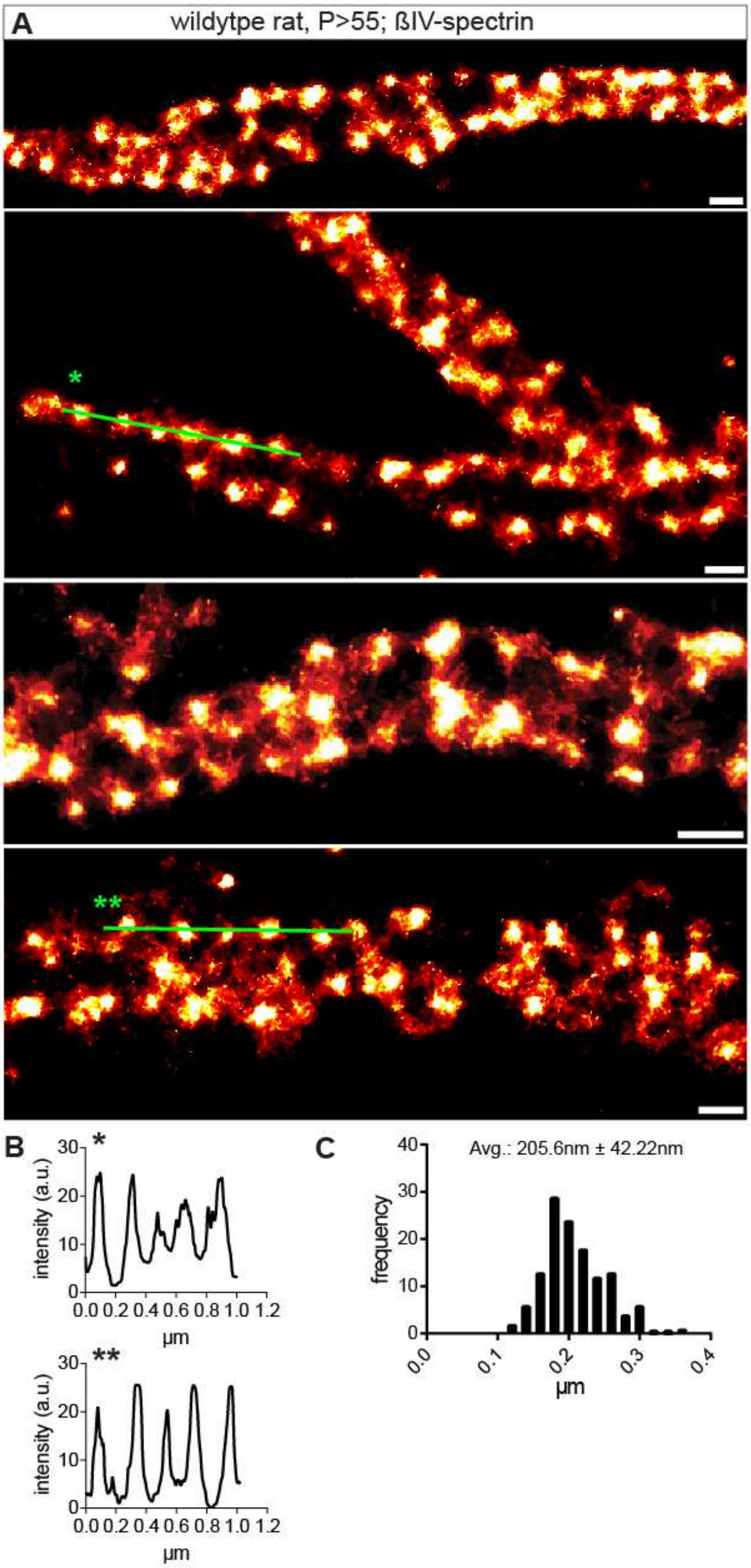

Supplementary Figure 2. RGC AIS nanostructure imaged by SMLM microscopy. (A) Different samples from whole mount retina AIS immunofluorescence against βIV-spectrin. AIS show lattice-like structures as also seen with STED, but due to labeling density and individual blinking behavior of fluorophores in SMLM, some structures appear brighter than others. Green lines indicate where line profiles in B were plotted. (B) Line profiles revealed periodic scaffold between fluorescent intensity peaks along the longitudinal axis of the AIS. (C) Size frequency histogram of a single interpeak indicating the period scaffold to be spaced at approximately 200 nm. Scale bar A = 1 µm, B = 500 nm; n = 5.

Supplementary Figure 3. Size frequency histograms of RGC AIS length during retinal development from P10 to P>55 (A-F) and after sensory deprivation (F+G). These graphs include all RGC AIS, irrespective of their synpo^{+} or synpo^{-} status. Analysis shows that the originally heterogeneous distribution of AIS length is significantly more homogeneous after P21, but remains at juvenile length distributions in both sensory deprivation conditions. (H) Summary of statistics analysis. Kruskal-Wallis test and Mann-Whitney t-test. *p ≤ 0.05, n = 6.

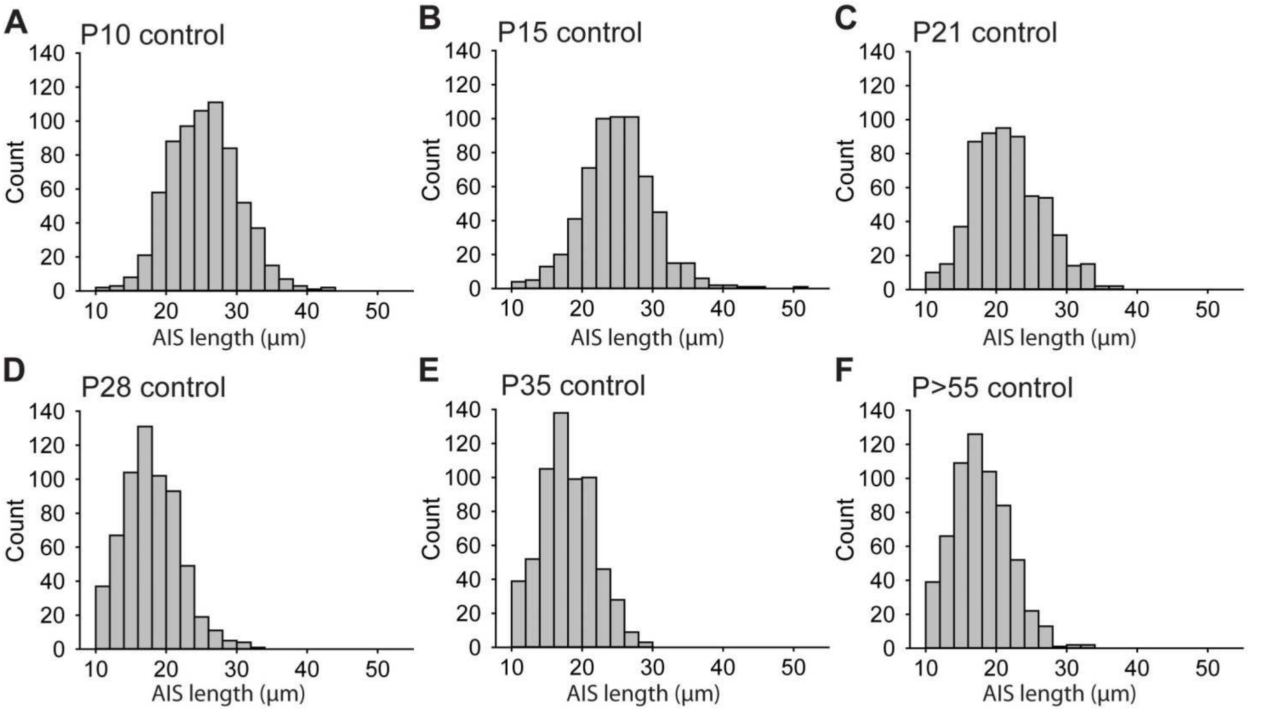

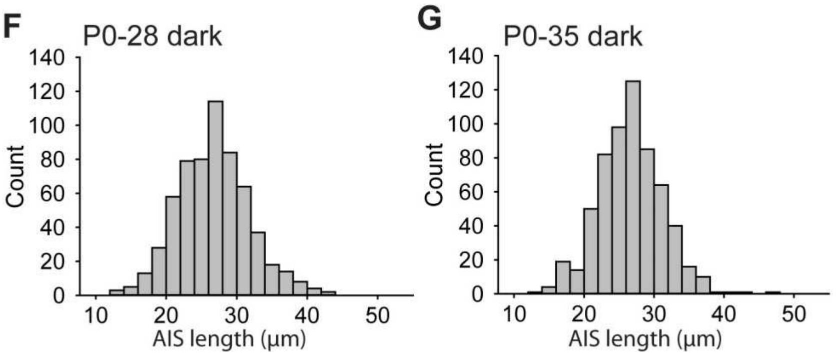

H

Comparison	P value
P10 ctrl vs. P15 ctrl	>0.9999
P10 ctrl vs P21/28/35/>55 ctrl	<0.5
P15 ctrl vs P21/28/35/>55 ctrl	<0.5
P21 ctrl vs P28/35/>55 ctrl	<0.5
P28 ctrl vs P35/>55 ctrl	>0.9999
P35 ctrl vs P>55 ctrl	>0.9999
P28 control vs. P0-28 dark	<0.5
P35 control vs. P0-35 dark	<0.5

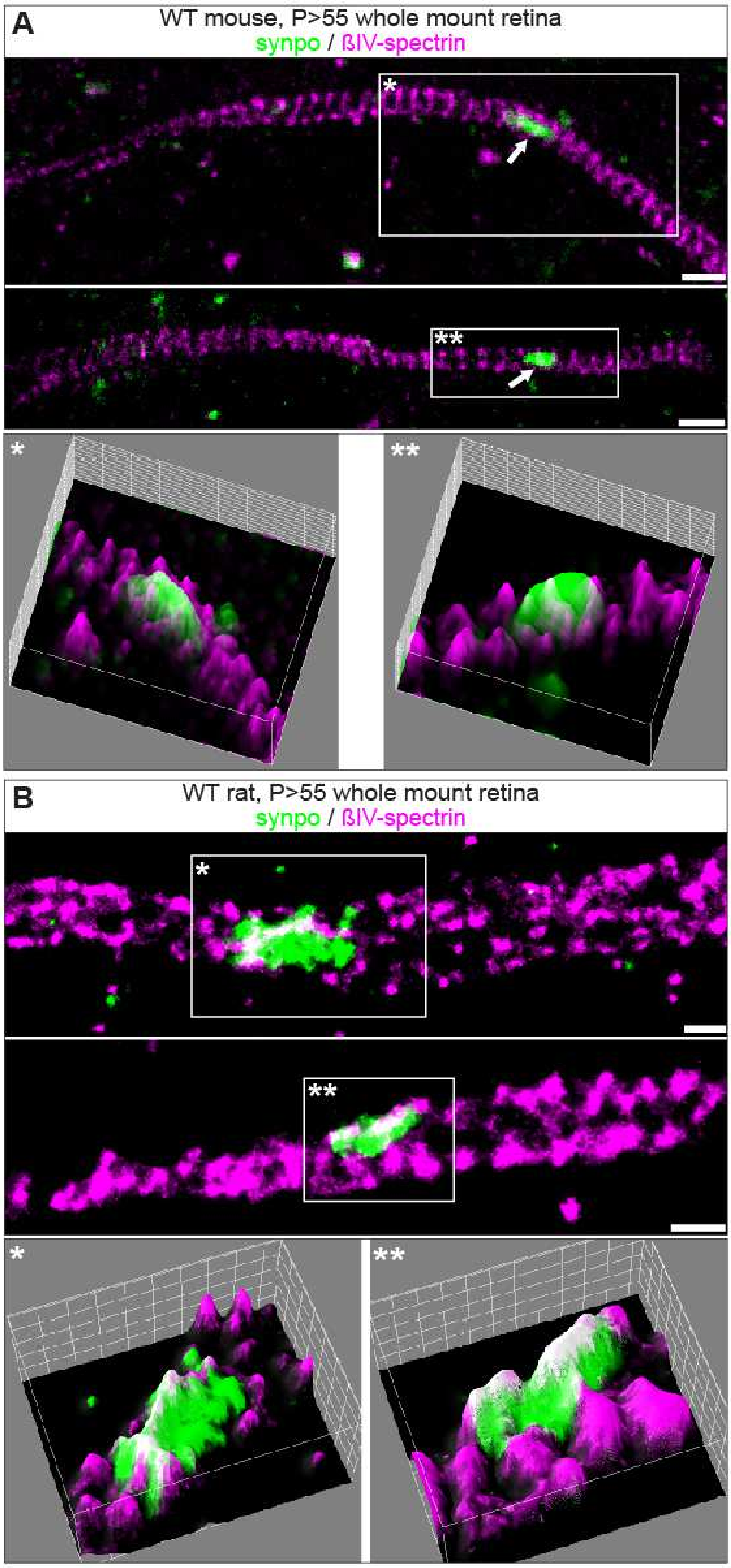

Supplementary Figure 4. Some synpo clusters associate with gaps in the AIS scaffold. (A) Upper and lower panel: STED images of a single RGC AIS with a synpo cluster (βIV-spectrin, magenta and synpo, green indicated by arrow). Boxes and asterisks indicate regions magnified in lower panel histograms. Here, synpo clusters appear in the center of the AIS but the scaffold is not interrupted by a gap. (B) Upper and lower panel: SMLM images of a single RGC AIS with a synpo cluster (βIV-spectrin, magenta and synpo, green). Boxes and asterisks indicate regions magnified in lower panel histograms. Synpo clusters sometimes appear in the center of the AIS in close vicinity to a gap in the spectrin scaffold, n = 5.

Supplementary Figure 5. Size frequency histograms of synpo+ RGC AIS length during retinal development from P10 to P>55 (A-F) and after sensory deprivation (F+G). Contrary to data shown in Supplementary Fig 2, synpo^{+} AIS are far more stable in length distribution regardless of developmental stage or sensory deprivation. (H) Summary of statistics analysis. One-way ANOVA and unpaired t-test. *p ≤ 0.05, n = 6.

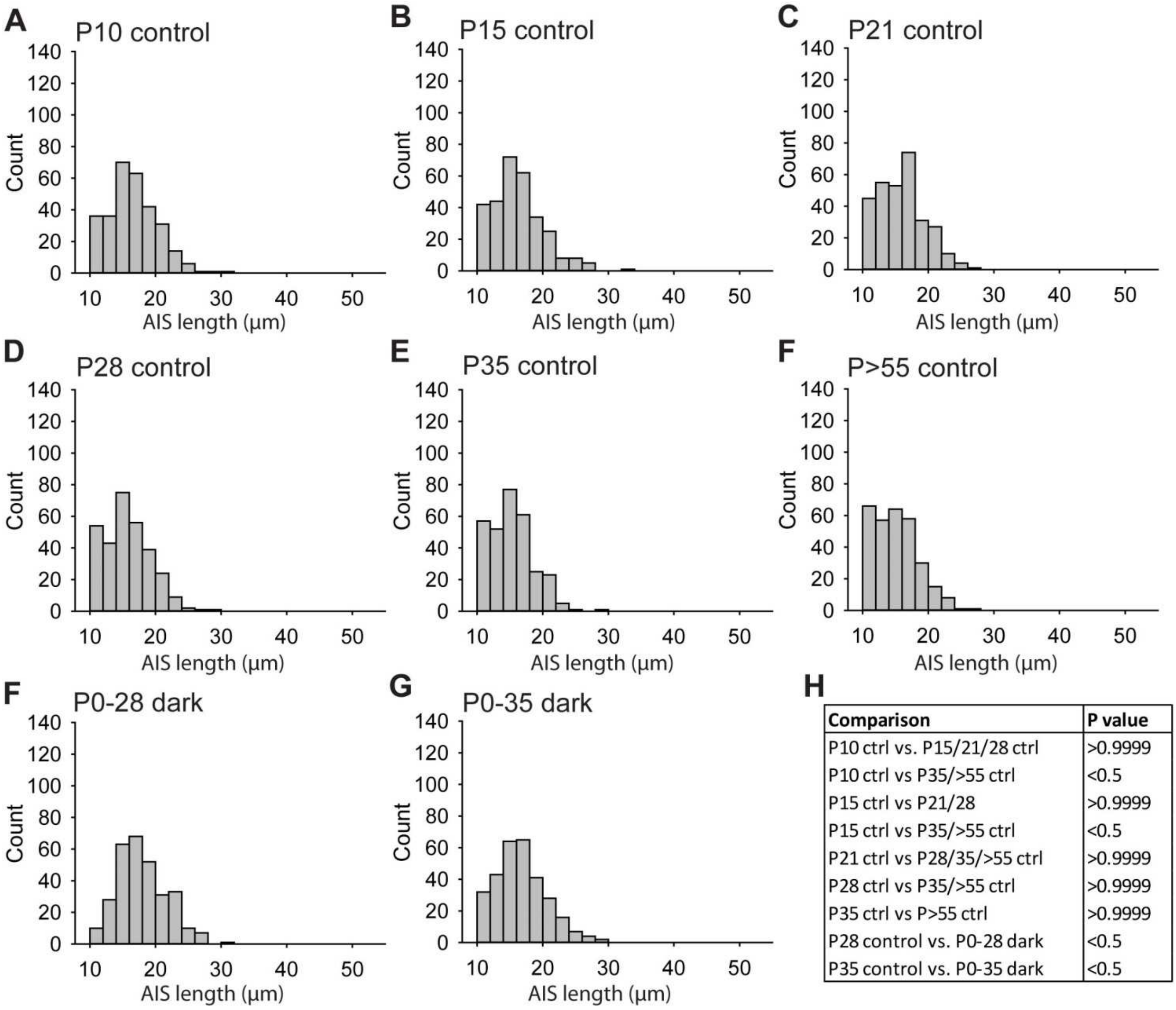

H

Comparison	P value
P10 ctrl vs. P15/21/28 ctrl	>0.9999
P10 ctrl vs P35/>55 ctrl	<0.5
P15 ctrl vs P21/28	>0.9999
P15 ctrl vs P35/>55 ctrl	<0.5
P21 ctrl vs P28/35/>55 ctrl	>0.9999
P28 ctrl vs P35/>55 ctrl	>0.9999
P35 ctrl vs P>55 ctrl	>0.9999
P28 control vs. P0-28 dark	<0.5
P35 control vs. P0-35 dark	<0.5

References (Supplement)

D'Este, E., Kamin, D., Velte, C., Gottfert, F., Simons, M., and Hell, S.W. (2016). Subcortical cytoskeleton periodicity throughout the nervous system. Sci Rep-Uk *6*.

Engelhardt, M., Vorwald, S., Sobotzik, J.M., Bennett, V., and Schultz, C. (2013). Ankyrin-B structurally defines terminal microdomains of peripheral somatosensory axons. Brain Struct Funct *218*, 1005-1016.

Gutzmann, A., Ergul, N., Grossmann, R., Schultz, C., Wahle, P., and Engelhardt, M. (2014). A period of structural plasticity at the axon initial segment in developing visual cortex. Front Neuroanat *8*, 11.

King, A.N., Manning, C.F., and Trimmer, J.S. (2014). A unique ion channel clustering domain on the axon initial segment of mammalian neurons. J Comp Neurol *522*, 2594-2608.

O'Brien, E.E., Greferath, U., and Fletcher, E.L. (2014). The effect of photoreceptor degeneration on ganglion cell morphology. J Comp Neurol *522*, 1155-1170.

Schedin-Weiss, S., Inoue, M., Hromadkova, L., Teranishi, Y., Yamamoto, N.G., Wiehager, B., Bogdanovic, N., Winblad, B., Sandebring-Matton, A., Frykman, S., *et al.* (2017). Monoamine oxidase B is elevated in Alzheimer disease neurons, is associated with gamma-secretase and regulates neuronal amyloid beta-peptide levels. Alzheimers Res Ther *9*.

Schlüter, A., Del Turco, D., Deller, T., Gutzmann, A., Schultz, C., and Engelhardt, M. (2017). Structural Plasticity of Synaptopodin in the Axon Initial Segment during Visual Cortex Development. Cereb Cortex *27*, 4662-4675.

Sun, W.Z., Li, N., and He, S.G. (2002). Large-scale morphological survey of mouse retinal ganglion cells. Journal of Comparative Neurology *451*, 115-126.
